# ACE: a probabilistic model for characterizing gene-level essentiality in CRISPR screens

**DOI:** 10.1186/s13059-021-02491-z

**Published:** 2021-09-23

**Authors:** Elizabeth R. Hutton, Christopher R. Vakoc, Adam Siepel

**Affiliations:** 1 Simons Center for Quantitative Biology, Cold Spring Harbor Laboratory, Cold Spring Harbor, NY USA; 2 Cold Spring Harbor Laboratory Cancer Center, Cold Spring Harbor, NY USA

**Keywords:** CRISPR, Essentiality, Statistical modeling, Gene addiction, Cancer

## Abstract

High-throughput CRISPR-Cas9 knockout screens are widely used to evaluate gene essentiality in cancer research. Here we introduce a probabilistic modeling framework, Analysis of CRISPR-based Essentiality (ACE), that accounts for multiple sources of variation in CRISPR-Cas9 screens and enables new statistical tests for essentiality. We show using simulations that ACE is effective at predicting both absolute and differential essentiality. When applied to publicly available data, ACE identifies known and novel candidates for genotype-specific essentiality, including RNA m^6^-A methyltransferases that exhibit enhanced essentiality in the presence of inactivating *TP53* mutations. ACE provides a robust framework for identifying genes responsive to subtype-specific therapeutic targeting.

**Supplementary Information:**

The online version contains supplementary material available at (10.1186/s13059-021-02491-z).

## Background

Pathogenic mutations and genotype-specific cancer liabilities can now be tested at unprecedented scales, owing to CRISPR-Cas9 (Clustered Regularly Interspaced Short Palindromic Repeats — CRISPR-associated protein 9) technology and large panels of diverse cell lines [1–10]. These technologies allow genetic vulnerabilities to be demonstrated through direct perturbation, rather than through indirect associations from population enrichment studies or gene expression levels. Furthermore, in comparison to previous screens based on RNA interference, CRISPR knockout screens offer substantially improved sensitivity and specificity, due to increased effectiveness at disrupting targeted elements and reduced off-target effects [11–13].

**Fig. 1:**
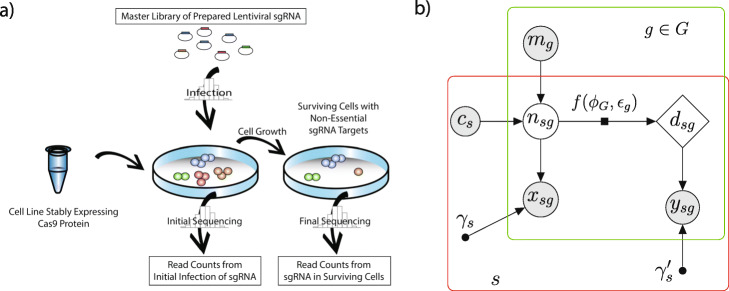
CRISPR-Cas9 Negative Selection Screen and Probabilistic Graphical Model. **a** The initial pool of Cas9-expressing cells is infected with a lentiviral sgRNA master library. The initial sgRNA abundances are measured by sequencing either the newly infected population of cells, the master library, or both. Cells are allowed to grow, and final sgRNA abundances are obtained by sequencing the surviving cells. **b** Graphical model illustrating how information is pooled across samples (*s*) and sgRNAs targeting each gene (*g*∈*G*) to infer each gene’s essentiality, *ϕ*_*G*_. From top-left to bottom-right, *m*_*g*_ represents the fraction of the master library consisting of sgRNA *g*; *c*_*s*_ represents the number of cells in sample *s* (estimated by the user); *n*_*sg*_ represents the number of cells initially infected with sgRNA *g* (such that *n*_*sg*_≤*c*_*s*_); *d*_*sg*_ represents the corresponding number of surviving cells after cell growth; and *x*_*sg*_ and *y*_*sg*_ represent the read counts obtained by sequencing *g* in the initial and surviving cells, respectively. The numbers of infected cells prior to (*n*_*sg*_) and following (*d*_*sg*_) growth are assumed to be related by the deterministic function *d*_*sg*_=*f*(*n*_*sg*_;*ϕ*_*G*_,*ε*_*g*_)=*n*_*sg*_(1−*ε*_*g*_*ϕ*_*G*_) (indicated by factor-graph notation). The efficiency *ε*_*g*_ is determined by logistic regression and the gene essentiality values *ϕ*_*G*_ are estimated by maximum likelihood (see Methods). The prior distribution for *n*_*sg*_ and the sampling distributions for *x*_*sg*_ and *y*_*sg*_ are assumed to be Poisson. The scaling factors *γ*_*s*_ and *γs*′ accommodate global properties of sequencing depth and cell growth, and are estimated in pre-processing (see the “ Methods” section). Shaded nodes represent variables observed in the data, unfilled nodes represent latent variables, and smaller solid circles represent free parameters

In a typical CRISPR-Cas9 screen (Fig. 1a), the artificially expressed Cas9 nuclease introduces a double-stranded break in native DNA, guided by homology with the separately infected single-strand guide RNA (sgRNA). The cell then repairs the double-stranded break, typically inserting or deleting several nucleotides at the cut site in the process. The resulting genetic lesion often disrupts the function of the targeted region. The genetically modified cell is allowed to proliferate for a series of divisions, after which the effects on cell growth are measured by sequencing the sgRNA present in the final surviving population of cells. Lethal sgRNA constructs that have disrupted sites critical for normal cell growth will be underrepresented in the final population in contrast to “nonessential” control sgRNAs. Moreover, differential essentiality can be detected from different levels of sgRNA depletion between samples. Hundreds of different cell lines have now been assayed in publicly available CRISPR knockout screens, offering an excellent opportunity to compare gene essentiality between panels of cancer cell lines according to their oncogenic mutations.

Within the general paradigm of a CRISPR-Cas9 screen, several variations of experimental design are possible. For example, many experiments evaluate the depletion of cells infected with lethal sgRNA by sequencing both the initial infected and the final population of cells [14–17]. In contrast, other experiments streamline the process by sequencing only the initial pool of sgRNA constructs prior to infection [9, 18, 19], and using the sgRNA abundances in this “master library” as a proxy for the initial frequencies. However, the sgRNA abundances in the master library typically vary by several orders of magnitude, and each biological replicate represents a separate infection into a new pool of cells, making this proxy imperfect. Overall, the variation in experimental design necessitates a flexible analytical framework that can robustly account for different sources of experimental error.

Through deliberate selection of target cell lines, a CRISPR-Cas9 screen can reveal genes that are essential only in a specific oncogenic context, for example, in the presence of a loss-of-function mutation of an important tumor suppressor such as *TP53*. These context-dependent essential genes — sometimes called “non-oncogene addictions” — are of particular interest because they may offer druggable therapeutic targets even when irreversible loss-of-function mutations to oncogenes are present [20]. A better understanding of genotype-specific gene essentiality has the potential to enable patient-specific therapeutics that are tailored to mutations present at the time of diagnosis. Non-oncogene addiction has recently achieved prominence, for example, in the first round of chemical inhibitors for RNA epigenetic modifiers soon to enter phase I trials [21].

However, statistical tests for identifying such context-dependent essentiality require special care. Not only are they subject to reduced power because they require contrasting one subset of the data with another, but they can yield spurious results if the “test” and “control” subsets are not adequately matched in other respects. Thus, rigorous testing frameworks are needed that efficiently make use of the data and control for such biases.

Many of the statistical tests that have been applied to CRISPR-Cas9 screens rely on summary statistics such as average read counts across samples, or log-fold changes of read counts between the initial and final populations of cells. However, these summary statistics are incomplete descriptions of the raw data, leading to inevitable loss of power. In addition, these tests can be difficult to adapt to alternative experimental designs. For example, methods such as BAGEL, CRISPhieRmix, and JACKS rely upon the log-fold change of read abundances [1, 2, 8–10, 22, 23], but these log-fold changes may be calculated with respect to read counts obtained from either the initial infected population of cells or the master library, with potentially substantial impacts on the power of the tests. Another issue is that some methods reduce the estimation of gene essentiality to a binary classification of ‘essential’ or ‘nonessential’ genes [2, 4, 24], which can blur the differences between weak and strong effects. Finally, most methods do not test directly for differential essentiality within sample subtypes (see Additional file 1: Figure S1 for a comparison of methods).

In this paper, we introduce the Analysis of CRISPR-based Essentiality (ACE), a flexible probabilistic method that directly estimates gene-level essentiality values by maximum likelihood, accommodates variable experimental designs, and enables rigorous likelihood-based tests of both absolute and differential essentiality. The ACE model describes each experimental phase of the CRISPR screen — the master library infection, and the initial and final sequencing — as a separate probabilistic process forming a hierarchical model. The modularity of our framework permits the analysis of a variety of CRISPR-screen data sets while accounting for differences in experimental design. We validate ACE using both simulations and published sets of essential and nonessential genes. We then apply the method to publicly available data from Achilles DepMap [9, 25] and demonstrate several compelling examples of differential essentiality in non-small cell lung cancer, including both known and novel cases.

## Results

### Model overview

ACE makes use of a hierarchical model design to integrate information at the sample, gene, and individual sgRNA levels, with separate parameters at each level (see Fig. 1). The model is structured to account separately for the major sources of uncertainty at each stage of the experimental process. In addition, if samples are partitioned into “test” and “control” groups, ACE can estimate gene essentiality within each sample group separately, allowing for the detection of differential essentiality (discussed further below).

As illustrated in Fig. 1b, the structure of the ACE model reflects the experimental design of a CRISPR-Cas9 screen. The initial number of infected cells *n*_*sg*_, for each sgRNA *g* and sample (replicate) *s*, is assumed to be Poisson-distributed with an expected value given by the relative frequency of guide *g* in the master library, *m*_*g*_, scaled by the number of cells, *c*_*s*_, infected in the assay. Estimates of both *m*_*g*_ and *c*_*s*_ are provided by the user. The final number of infected cells, *d*_*sg*_, is then assumed to be given by a scaled version of the initial number, *d*_*sg*_=*n*_*sg*_(1−*ε*_*g*_*ϕ*_*G*_), where *ε*_*g*_∈[0,1] denotes the editing efficiency of sgRNA *g* and *ϕ*_*G*_≤1 denotes the essentiality of gene *G*. These initial and final numbers of infected cells are not observed directly (they are latent variables) but are indirectly sampled via the sequencing process, resulting in observed read counts of *x*_*sg*_ and *y*_*sg*_, respectively, which are provided by the user as inputs to the inference procedure. We assume Poisson-distributed read counts *x*_*sg*_ and *y*_*sg*_, and sum over possible values of the latent variable *n*_*sg*_ in the likelihood calculation (see the “Methods” section). Notably, the combination of the Poisson prior and the Poisson sampling distribution adequately captures the observed overdispersion in the read count data (see the “Discussion” section and Additional file 1: Figure S2). Independence is assumed across samples and replicates. The free parameters of the model are estimated by numerical maximization of the likelihood function (see Methods). The ACE software reports estimates of not only the essentiality of each gene, *ϕ*_*G*_, but also the efficiency of each sgRNA, *ε*_*g*_.

A value of *ϕ*_*G*_=1 represents complete essentiality (driving *d*_*sg*_=*n*_*sg*_(1−*ε*_*g*_*ϕ*_*G*_) to zero in the case of efficient editing, *ε*_*g*_=1), whereas a value of *ϕ*_*G*_=0 represents complete nonessentiality (allowing *d*_*sg*_=*n*_*sg*_ even when *ε*_*g*_>0). Notably, however, our definition of *ϕ*_*G*_ also allows for negative values, reflecting increased cell growth after inactivation of gene *G*. Such an increase might occur, for example, if a tumor suppressor gene were deactivated by guide RNAs. In practice, negative estimates of *ϕ*_*G*_ are fairly frequently observed, either by random chance or from true increases in cell growth.

To pool information across sgRNAs and ensure identifiability of *ε*_*g*_ and *ϕ*_*G*_, we determine *ε*_*g*_ by logistic regression based on a user-defined set of features, and treat the regression coefficients only as free parameters (see the “Methods” section). The efficiency of a class of sgRNA has been shown to be strongly correlated with a number of genomic features, including local GC content, proximity to heterochromatin, number of base mismatches, and abundance of off-target sites [18, 26–29]. However, for simplicity and efficiency, we have used only the GC content of the sgRNA (one of the strongest predictors of efficiency) in our initial implementation. Nevertheless, ACE can easily be extended to consider additional features.

The two sample-specific scaling factors, *γ*_*s*_ and *γs*′, determine the relationships between the numbers of infected cells and the expected sgRNA counts in the initial and final data sets, respectively. These parameters apply globally to all sgRNAs and all genes. Rather than including these parameters in the numerical optimization of the likelihood function, we find it adequate to estimate them in preprocessing based on a median-of-ratios normalization, as used in the widely used differential expression analysis tool, *DESeq2* [5].

Notably, *γ*_*s*_ is primarily a reflection of sequencing read depth, whereas *γs*′ is determined by both read depth and sample-specific factors in cell growth (see the “Methods” section). In the case where the bulk distribution is strongly influenced by essential genes, as when a library has a large number of essential targets, *γs*′ can alternatively be estimated from designated negative controls.

#### Differential essentiality

In the case of differential essentiality, ACE compares two (log) likelihoods for each gene *G*: a likelihood maximized under the constraint that the essentiality, *ϕ*_*G*_, is the same in designated “test” and “control” sample categories, and a likelihood that allows for different values of *ϕ*_*G*_ in these two categories. These sample categories can be selected by the user to examine differences, for example, between cell lines with and without mutations in *KRAS*, or between lung- and liver-derived sample panels. The two per-gene likelihoods are then compared in a likelihood ratio test, with significance assessed by comparison with an empirical null distribution based on simulated data (see the “Methods” section). In this way, ACE performs a rigorous test of the alternative hypothesis that gene *G* exhibits different degrees of essentiality in the test and control categories against a null hypothesis of equal essentiality.

### Simulation study

To evaluate the ability of ACE to infer sgRNA-, sample-, and gene-specific parameters, we devised a simulation method to generate artificial CRISPR-screen data sets. To ensure that our simulated data resemble data from real CRISPR screens as closely as possible, our simulator, called empiriCRISPR, mimics the variation in read counts observed in a reference experiment and makes minimal assumptions about the processes by which the data are generated (see the “Methods” section and Additional file 1: Figure S3). Importantly, empiriCRISPR is fully independent of the probabilistic model and parameter inference used in ACE.

#### Benchmarking of absolute essentiality predictions

**Fig. 2:**
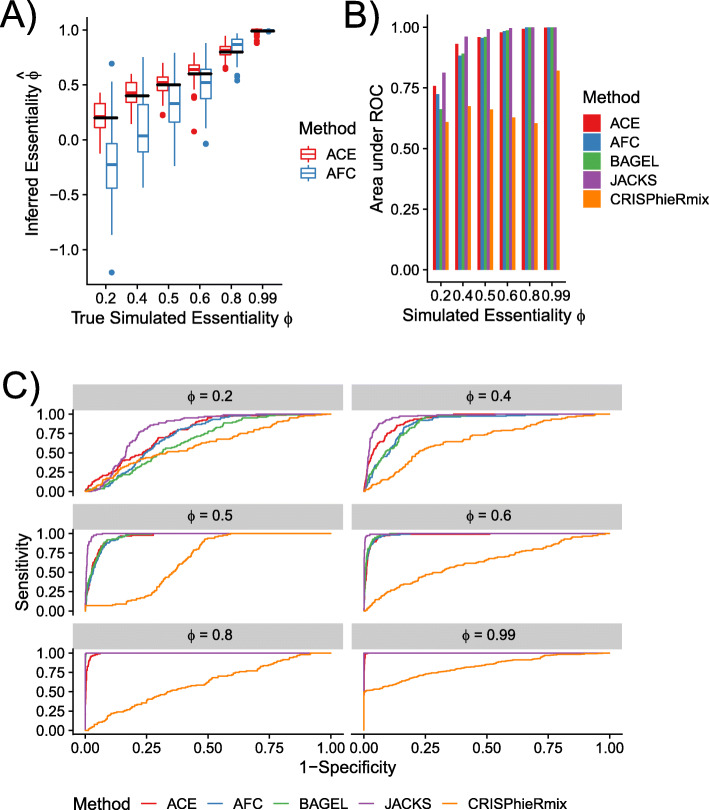
Detection of Absolute Essentiality in Simulated Data. **A** Inferred essentiality values ($\widehat \phi $) for 300 genes simulated in three replicates at each essentiality value shown (*ϕ*). Simulated data sets also included 3150 nonessential genes (*ϕ*_*G*_=0; not shown), 300 of which were provided to each method as negative controls. Black horizontal bars indicate true values ($\widehat \phi = \phi $). **B** Performance in binary classification (essential vs. nonessential) of simulations of 300 “essential” genes at various values of *ϕ*_*G*_ and 300 “nonessential” genes. Performance reported as the Area under the Receiver Operating Characteristic (ROC) curve. Only initial and final read counts from the simulation were used for all methods. **C** Full ROC curves for each of the simulated essentiality values. “ACE” — our probabilistic method (thresholded on log likelihood ratios), “AFC” — method based on average fold changes in sgRNA abundance (thresholded on *z*-scores; see the “Methods” section). “BAGEL” — Bayesian Analysis of Gene Essentiality [2] (thresholded on reported Bayes Factors); “JACKS” — Joint analysis of CRISPR/Cas9 knockout Screens [8] (thresholded on reported *p*-values). “CRISPhieRmix” — hierarchical mixture model [23] (thresholded on reported essential gene FDR)

Using empiriCRISPR, we generated data sets of 5250 genes in which the essentiality of each gene ranged from complete nonessentiality (*ϕ*_*G*_=0) to complete essentiality (*ϕ*_*G*_=1). To ensure a fair comparison with methods that rely on a median-based normalization method, we took care to include an adequate number of nonessential genes in each data set, simulating 3,150 genes with *ϕ*_*G*_=0 (60% of all genes). The remaining 40% of genes consisted of 300 genes at each of several intermediate values of *ϕ*_*G*_ and 600 genes at *ϕ*_*G*_=0.99 (see Fig. 2A). For each choice of simulation parameters, three replicates were simulated with four sgRNAs targeting each gene and an average of 300 reads per sgRNA. As a template data set for empiriCRISPR, we used a collection of previously published genome-wide CRISPR screens in which both the master library and initial infected sgRNA abundances were sequenced along with the depleted samples [16]. The master library was generated through random selection of sgRNA from the template screen, followed by simulation of read counts according to the patterns of variation observed in the template.

We first compared ACE’s estimates of essentiality with an estimator based on the average fold change (AFC) in read counts (see the “Methods” section), which has been used in several previous studies [17, 30] (see Fig. 2A). We found that the ACE estimates are approximately unbiased and exhibit relatively low variance (see also Additional file 1: Figure S4). By contrast, the AFC-based estimates display a downward bias, particularly at low simulated values of *ϕ*_*G*_, and show substantially greater variance. Notably, the simple AFC-based method often yields negative estimates of *ϕ*_*G*_ even when the true *ϕ*_*G*_ is positive.

Many methods do not provide a numerical estimate of the essentiality of a gene, but instead focus on a binary classification of genes into “essential” and “nonessential” categories. To compare ACE with these methods, we simulated data sets containing 300 genes at each of several values of *ϕ*_*G*_, together with 3150 nonessential genes. For each of these experiments, we then compared the binary classification performance of ACE with BAGEL [1], JACKS [8], CRISPhieRmix [23], and a classifier based on simply thresholding the AFC statistic. We summarized the performance of all methods using receiver operating characteristic (ROC) curves and the area under the ROC curve (AUC) metric (Fig. 2B and C). We found that most methods performed similarly well at high values of *ϕ*_*G*_, but in the harder scenarios where *ϕ*_*G*_ takes moderate to low values (*ϕ*_*G*_<0.5), JACKS and ACE performed significantly better than the others. JACKS, however, slightly outperformed ACE on this task, apparently because it makes particularly efficient use of the negative controls in defining a null distribution. CRISPhieRmix performed substantially worse than the other methods in this test.

#### Benchmarking of differential essentiality predictions

To test the detection of differential essentiality, we simulated CRISPR-screen data in two samples (“test” and “control”) that included 300 genes essential only in the “test” sample with several different choices of $\phi _{G_{test}}$ (see the “Methods” section). To represent uniformly essential genes, both samples also included 300 genes at strong (*ϕ*_*G*_=0.99) and 300 genes at moderate (*ϕ*_*G*_=0.5) essentiality, as well as a large collection (3150) of nonessential genes (*ϕ*_*G*_=0). Similar to the previous tests, we compared the performance of ACE with BAGEL, JACKS, CRISPhieRmix, and a simple AFC-based method in the detection of the differentially essential genes. Because the other methods do not support direct tests for differential essentiality, we devised heuristic test statistics based on the results of two separate tests for essentiality. After some experimentation, we settled on using the ratio of *p*-values for the test and control panels for JACKS, as there is only one “sample type” in our simulation. For CRISPhieRmix, we used the ratio of predicted false discovery rates between test and control panels, and for BAGEL, we used the absolute difference in the Bayes Factors for the test and control panels (see the “Methods” section).

**Fig. 3:**
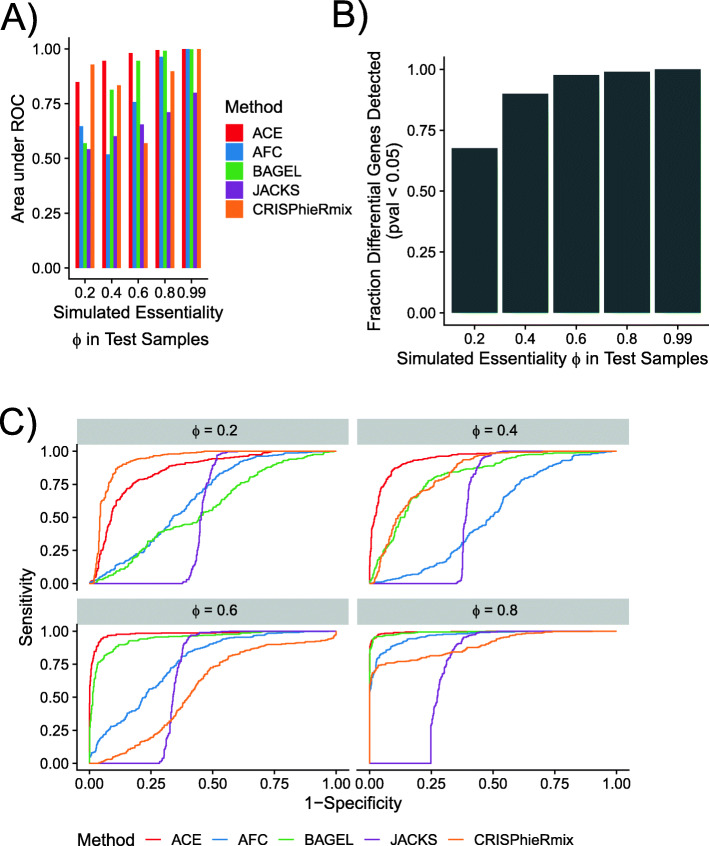
Detection of differential essentiality in simulated data. **A** Performance in binary classification of differential essentiality for 300 genes that were “essential” (at various levels of simulated essentiality *ϕ*) in “test” samples and “nonessential” (*ϕ*=0) in “control” samples. Both samples also included 300 genes at strong (*ϕ*_*G*_=0.99) and 300 genes at moderate (*ϕ*_*G*_=0.5) essentiality, as well as a large collection (3150) of nonessential genes (*ϕ*_*G*_=0). 300 genes from each of these uniformly essential sets were used as negative controls. **B** Fractions of differentially essential genes correctly detected by ACE (at a Bonferroni-corrected empirical *p* < 0.05) at various values of simulated essentiality *ϕ*. **C** Full ROC curves for each of the simulated differential essentiality levels. In all cases, three replicates of the control and test samples were simulated for each value of *ϕ*. Methods compared are as described in Fig. 2

We found that ACE’s likelihood ratio test for differential essentiality did result in substantially better power than the competing methods, on average (Fig. 3). Most methods had reasonably good power when $\phi _{G_{test}}$ was large (≥0.8) with JACKS and CRISPhieRmix performing somewhat more poorly than the others, but at values of $\phi _{G_{test}} \leq 0.6$, ACE showed a clear advantage, except for the weakest signal of differential essentiality ($\phi _{G_{test}} = 0.2$), at which it was surpassed by CRISPhieRmix. Even when $\phi _{G_{test}} = 0.2$, ACE was able to detect 67% of differentially essential genes (Fig. 3B).

### Analysis of real data from CRISPR screens

#### Identification of essential genes

We next sought to evaluate the performance of ACE in detecting previously identified essential genes from real data. We focused on 688 genes that have been classified as essential in at least two previous studies [1–3], based on both RNAi and CRISPR screens [19]. For negative controls, we used 634 genes that are not widely expressed according to public RNA-seq data (see Methods). We used ACE to evaluate the essentiality of both gene sets using the CRISPR-screen data from Achilles DepMap. Notably, this data set includes sequence data representing only the sgRNA master library and the post-depletion sgRNA abundances. To ensure a similar genetic background between samples, we focused on 92 unique cell lines that all originate from lung adenocarcinoma, a subtype of non-small cell lung cancer (NSCLC).

**Fig. 4:**
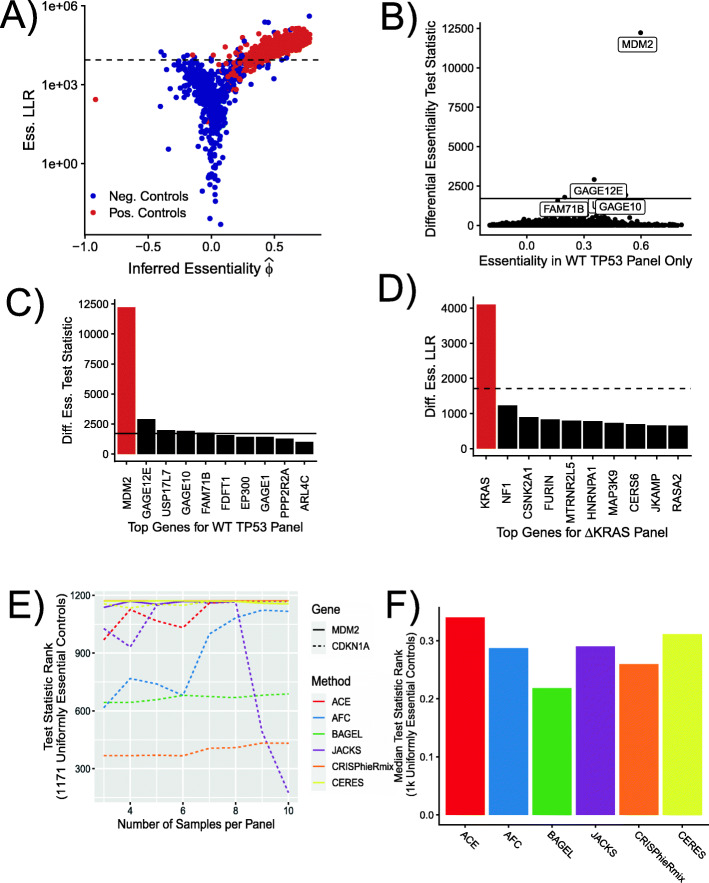
Identification of known genotype-specific essentiality. **A** Estimates of essentiality per gene ($\widehat \phi $; *x*-axis) and essentiality log likelihood ratios (Ess. LLR; *y*-axis) from ACE based on genome-wide CRISPR-screen data from the Achilles DepMap Project. Results are shown for 688 “essential” genes (Pos. Controls; *red*) identified in references [1–3] and 634 nonessential genes (Neg. Controls; *blue*; see the “Methods” section for details). The LLR metrics are relative to a null hypothesis of $\widehat \phi =0$. **B** Estimated essentiality per gene in wild-type *TP53* (*ϕ*; *x*-axis) vs. log likelihood ratio (LLR) for differential essentiality in wild-type vs. mutant *TP53* based on DepMap data for NSCLC Adenocarcinoma [9]. Several high-scoring genes are highlighted with labels, including *MDM2* (see text). **C** Top 10 genes identified as differentially essential in NSCLC samples having wildtype *TP53*, ordered by differential essentiality log likelihood ratio (Diff. Ess. LLR). **D** Top 10 genes identified as differentially essential in NSCLC samples having mutant *KRAS*. In panels **A**–**D**, the horizontal dashed lines indicate Bonferroni-corrected empirical *p*-values of 0.05. Genes highlighted in red in panels **C** and **D** are discussed in the text. **E** Ranking of positive (*CDKN1A*) and negative (*MDM2*) regulators of *TP53* by differential essentiality test statistic across all prediction methods. Ranking is relative to 1171 uniformly essential or nonessential genes from our previously described negative and positive control sets; a subset of 50 from each of these sets were provided to methods as annotated controls. An illustration of *MDM2* alone is shown in Additional file 1: Fig. S9. **F** Median ranking of 19 differentially expressed genes and their 14 associated genetic backgrounds across five tissue types from DepMap’s Project Achilles. Ranking is relative to 500 uniformly essential and 500 uniformly nonessential genes from our previously described negative and positive control sets. Method performance by gene and tissue type is shown in Additional file 1: Figs. S11 and S12. Results from a one-sided test using expected direction of essentiality are shown in Additional file 1: Fig. S13

Considering the many differences in methods and data sets, we found that ACE’s characterization of these genes was fairly concordant with previous results. The genes previously identified as essential (positive controls) yielded both clearly elevated estimates of essentiality ($\widehat \phi $) and clearly elevated log likelihood ratios (LLRs) in a test for non-zero essentiality relative to the negative controls (Fig. 4A). At a LLR threshold defined such that only 5% of the negative controls exceeded it (i.e., with empirical *p*=0.05), 90.5% of the positive controls were predicted to be essential. In an ROC analysis based on the same data, ACE was able to distinguish the positive and negative controls with high accuracy (AUC = 0.97; see Additional file 1: Figure S5). The outliers in the negative control population appear to be mis-classified as nonessential due to low expression levels, despite that they may actually have essential (e.g., *DUX4*) or growth-suppressing activity (e.g., *POU1F1* [31]).

#### Identification of differentially essential genes

To evaluate ACE’s ability to identify differential essentiality with real data, we applied it to genes evaluated in liver and lung tissue in the DepMap database. For our set of uniformly essential genes, we selected both a “uniformly active” set of 698 genes robustly expressed across lung- and liver-derived samples, and a “uniformly silent” set of 594 genes showing little or no evidence of expression across samples (see the “Methods” section). For the target set of differentially essential genes, we used as a proxy genes that exhibited a substantial difference in median expression (≥1.5 TPM) between tissue panels, excluding genes that were highly expressed (>5 TPM) in both panels (Additional file 1: Figure S6). We then predicted differential essentiality between the liver and lung samples using each of the computational methods under evaluation (Figures S7 and S8).

We found that ACE performed roughly as well as the best methods on this task (AUC = 0.701, better than all but CRISPhieRmix [0.709] and the AFC-based method [0.705]). However, all methods performed, at best, only modestly well on this benchmark, likely owing to the disparity between gene expression levels and gene essentiality as evaluated by a knockout phenotype. The main outlier in this analysis was CERES, which showed considerably poorer performance than the other methods, possibly because it was optimized for uniformly essential and nonessential genes across all DepMap samples.

#### Detection of known cases of genotype-specific essentiality

One possible application of ACE’s test for differential essentiality is to identify significant differences in essentiality between distinct genetic backgrounds. For example, cases of synthetic lethality may appear as genes that are essential in test samples in which another gene is mutated and nonessential in control samples in which that gene is intact. Conversely, other genes may be found to be essential in the presence of the wildtype but not the mutant genotype. To explore this possibility, we contrasted cell lines annotated by the Cancer Cell Line Encyclopedia (CCLE) with variants of two well-known cancer-related genes, the tumor suppressor *TP53* and the proto-oncogene *KRAS* (see the “Methods” section) [32, 33].

We restricted ourselves to NSCLC samples in these cases, to avoid spurious signals unrelated to the mutations of interest. Moreover, we structured our test specifically to identify genes that were significantly more (rather than less) essential in the test samples than in the control samples. As in the previous analysis, we used data from Achilles DepMap [9, 25].

In the case of *TP53*, we first defined the test samples as ones with “wildtype” copies of *TP53*, with no annotated mutations, and the control samples as ones in which *TP53* has “damaging” mutations (denoted here as *ΔTP53*; see the “Methods” section). This test for a wildtype-dependent increase in essentiality yielded a clear outlier: the gene *MDM2* (Fig. 4B and C). Cell lines with wildtype *TP53* require an intact copy of *MDM2* to negatively regulate the TP53 protein and allow for cell proliferation [34, 35]. The dependence of cell lines with wildtype *TP* on functional *MDM2* observed by ACE therefore provides support for the biological validity of our test for differential essentiality.

In the case of *KRAS*, which becomes oncogenic in the presence of gain-of-function mutations and is known to drive aggressive tumorigenesis [36], we defined the samples in the opposite way: with test samples containing a missense mutation in *KRAS* (*ΔKRAS*), the majority of which are activating gain-of-function mutations [37], and control samples with no annotated *KRAS* SNP. Samples containing wildtype *TP53* were additionally excluded to remove the signal from *MDM2*. As predicted, the *KRAS* gene itself emerged as having, by far, the strongest signal for a mutation-dependent increase in essentiality (Fig. 4D), further validating our method.

To compare computational methods in this setting, we applied all methods to *MDM2* and *CDKN1A*, a negative and a positive regulator of *TP53*, respectively [38]. Again, we tested test samples having “wildtype” *TP53* against control samples having *ΔTP53*. Moreover, we examined the performance of all methods as a function of the number of samples per panel, quantifying performance using the ranking of each gene relative to 1171 uniformly positive and negative control genes. ACE consistently ranked both genes highly for differential essentiality, both in the low-sample panel regime, and as more samples were included (Fig. 4E and Additional file 1: Figure S9). The other methods consistently ranked *MDM2* highly, but most methods performed more poorly in the case of *CDKN1A*, likely because they are not designed to detect the ‘negative essentiality’ associated with the loss of positive regulator.

We found this trend holds across multiple tissues and genes (Fig. 4F), with ACE consistently ranking known differentially essential genes higher than uniform controls. In this case, we analyzed 19 known instances of differentially essential genes in cell-line panels derived from five different tissue types (see Additional file 1: Figures S10-S13). Even in the relatively noisy regime of three samples per panel, ACE was able to detect additional signals of differential essentiality.

#### Test for non-oncogene addictions in *TP53* mutants

**Fig. 5:**
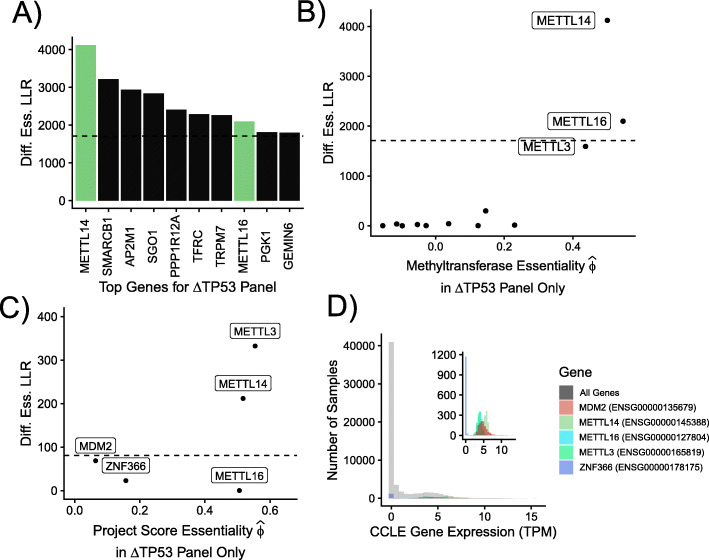
RNA Methyltransferase Genes Associated with *TP53*. **A** Top 10 genes (by differential essentiality log-likelihood ratio) showing increased essentiality in NSCLC cell lines that harbor “damaging” mutations in *TP53* (*ΔTP53*) according to the CCLE, suggesting possible non-oncogene addictions. The RNA m^6^-A methyltransferases *METTL14* and *METTL16* (*green*) are of particular interest (see text). **B** Estimated essentiality of RNA methyltransferase genes in *ΔTP53* samples (*x*-axis) vs. log likelihood ratio in *ΔTP53* vs. wild-type *TP53* samples (*y*-axis). **C** Evaluation of methyltransferase gene essentiality in Project Score CRISPR screens. Only two unique lung-tissue-derived cell lines (3 replicates each) carry wildtype *TP53* in the Project Score database, so only two cell lines with a “damaging” mutation in *TP53* were selected for comparison using ACE. The cell lines chosen were based on our analysis in the Broad’s Project Achilles, but the data used is generated by screening with a different CRISPR library by the Sanger Institute. The dashed line indicates the empirical p-value cutoff for *p*<0.05 based on negative controls. **D** Gene expression data from the CCLE, processed by the Achilles DepMap, for the *ΔTP53* cell line panel highlighting the differentially essential methyltransferase genes from panel **(B)**. *MDM2* is shown as a positive control and nonessential *ZNF366* is shown as a negative control. The distribution of all genes is shown in gray

Finally, we swapped the “test” and “control” samples for *TP53* — using *ΔTP53* samples for the “test” and wildtype samples for the “control” — with the goal of discovering novel non-oncogene addictions. As discussed above, such addictions may suggest new drug targets in cases of undruggable loss-of-function mutations. This test of increased essentiality in the presence of damaging *TP53* mutations identified several candidate genes (Fig. 5A and Additional file 1: Figure S14).

Notably, the top candidates included two genes encoding RNA m^6^-A methyltransferases, *METTL14*, and *METTL16*. A third gene, *METTL3*, encoding the heterodimeric partner of *METTL14* also scored highly for differential essentiality (Fig. 5B). When examined in CRISPR knockout screens performed by the Sanger Institute’s Project Score, using a different master sgRNA library, both *METTL14* and *METTL3* were found to be essential only in the presence of “damaged” *TP53* in lung samples (Fig. 5C). All three methyltransferases are expressed at a moderate to high level across the *TP53*-mutant cell line panel, according to CCLE expression data (Fig. 5D and Additional file 1: Fig. S15) [25, 32, 33].

Several studies have noted CRISPR sgRNA cutting in multiple regions can reduce cell proliferation, regardless of the essentiality of the sites targeted. To ensure that the signatures of essentiality in *METTL3*, *METTL14*, and *METTL16* are not merely reflective of changes in copy number, we compared gene duplications between mutant and wildtype *TP53* sample panels (Additional file 1: Fig. S16). Copy number variation was similar between the two panels, suggesting that differences in gene essentiality are driven by biological activity of the genes themselves. In addition, flanking genomic regions of *METTL14* and *METTL16* do not contain known oncogenic drivers, implying that CRISPR-induced lesions are unlikely to be simultaneously influencing a nearby essential gene (Additional file 1: Fig. S17). An additional analysis of Project Achilles liver samples revealed no significant differential essentiality, with little difference in absolute essentiality observed between samples with “damaged” or wildtype *TP53* (Additional file 1: Fig. S18). Overall, our findings suggest that *METTL3*, *METTL14*, and *METTL16* are promising candidates for non-oncogene addiction in the absence of functional *TP53* in lung NSCLC Adenocarcinoma.

## Discussion

In this paper, we have introduced a new probabilistic modeling and inference method, called ACE, for detecting essential genes and estimating levels of essentiality from CRISPR negative selection screens. As we have shown, ACE uses a hierarchical model to account for the uncertainty associated with each major stage of a CRISPR screen and enable maximum-likelihood estimation of gene-level essentiality. It supports likelihood ratio tests for both absolute and differential essentiality. In comparison with other available computational methods, ACE appears to be particularly advantageous in cases of differential essentiality, moderate-to-weak essentiality, or limited numbers of samples. In addition, while we have not highlighted this feature in our analyses, the hierarchical structure of the ACE model makes it particularly well suited for integrating data across experiments, or across groups of genes. We anticipate that ACE’s performance can be further improved by incorporating state-of-the-art models for sgRNA editing efficiency into the method.

While the number of known essential genes is growing, simulations remain critical in providing an objective gold standard to evaluate the performance of computational methods. To test ACE and compare it with other available methods, we developed a new simulator, called empiriCRISPR. Our strategy with empiriCRISPR was not to generate simulated data according to a process-based model — which would tend not to account for all of the sources of noise in real data, and would be difficult to define without heavily overlapping the assumptions of ACE itself — but instead to mimic the empirical properties of real data sets as closely as possible. In this way, we were able to generate ‘authentic-looking’ data sets of arbitrary size with known sets of essential genes at known levels of essentiality. We showed that ACE performs quite well on these data sets despite their realistic ‘noise’ characteristics.

To our knowledge, ACE is the first method to support a direct likelihood-based test for differential essentiality between designated ‘test’ and ‘control’ sample panels. This test can be useful in a variety of applications, including tests for differences in essentiality in the presence of mutant and wild-type versions of known cancer genes. In comparison to indirect tests for differential essentiality — derived from separate analyses of “test” and “control” panels — ACE appears to offer improved power according to our simulation study (see Fig. 3). Notably, it is straightforward to define one-sided variants of this test to detect *increased* or *decreased* essentiality in the “test” relative to “control” panels. The framework also extends naturally to tests of more complex hypotheses (e.g., test essentiality >0 and control essentiality ≤0). These should all be well-powered tests, provided they are applied to sufficiently abundant data for the desirable asymptotic properties of likelihood ratio tests to become apparent.

Whereas most current statistical models for sequencing read counts allow for overdispersed counts, typically through the use of a negative binomial distribution, we settled on the use of the simpler Poisson distribution in this work. We initially experimented with a negative binomial distribution for read counts, allowing the gene-wise dispersion parameter to vary as a function of the mean, as in DEseq2 [5]. However, we found this version of the model computationally expensive to optimize and prone to over-fitting. The Poisson version of the model is simpler, more robust, and easier to fit to data. Moreover, our integration over possible values of the Poisson mean appears to be adequate to account for the major effects of overdispersion. In the end, we observe comparable performance between ACE and methods that use more sophisticated variance estimation methods.

As illustrated by the major revisions of “core essential gene” sets over the past 10 years, the definition of “essential genes” is inevitably somewhat subjective and arbitrary. In reality, essentiality occurs on a spectrum and depends on the particular genetic background, cell-type, and conditions tested. As a result, candidate “essential” genes require carefully designed experiments for validation, and researchers should be cautious about relying upon published sets of genes labeled “essential”. Estimates of *ϕ*_*G*_ in ACE are also limited in that they reflect an “average” essentiality across all samples analyzed, so users must take care to choose a panel of samples that ensures a level of diversity appropriate for the biological question of interest. For example, a diverse collection of tissue samples or cell lines may be most suitable for the identification of universally essential genes, whereas a set of patient-derived tissue samples may be best for personalized tumor therapeutics. Notably, because it is a relative metric, differential essentiality may in some cases generalize better than absolute essentiality. As illustrated in Additional file 1: Fig. S7, the imperfect correlation of gene expression with its knockout phenotype restricts the identification of truly differential genes to data such as CRISPR screens. We anticipate that findings of differential essentiality may be of interest to researchers exploring genetic vulnerabilities even in other tissues and cancer subtypes.

We have focused in this work on differential essentiality between previously defined groups of samples, but it is worth noting that ACE could be adapted to identify sample subgroups of interest de novo on the basis of differential essentiality. For example, one could iterate over all possible bipartitions of a set of samples and identify subgroups exhibiting the strongest signals of differential essentiality. If this approach proved too computationally intensive, candidate bipartitions could be identified based on pre-computed individual gene estimates of essentiality. We anticipate that subtype discovery may be a promising area for future applications of ACE.

Among the genotype-dependent differentially essential genes identified by ACE, we found the RNA methyltransferase genes *METTL3*, *METTL14*, and *METTL16* to be particularly interesting. RNA m^6^-A modifications have recently been found to be critical in the maintenance of leukemic cell growth [39]. These genes have been previously implicated in other cancer types, and RNA m^6^-A methyltransferases in general have been a topic of recent interest in the new field of cancer epitranscriptomics, with several phase I trials targeting METTL3 due to begin in 2021 [21]. METTL3, in particular, was recently identified as interacting with *TP53* mRNA and inducing drug resistance in a biochemical assay in SW48 colon cancer cells [40]. Another study found *METTL3* expression to be essential in NSCLC adenocarcinoma, although only two cell types were examined [41]. *METTL3* mRNA knockdown with siRNA has also been shown, by M6A-seq, to result in an enrichment of methylation changes in genes in the p53-signaling pathway [42]. Finally, patients with genetic alterations of m^6^-A regulatory genes have been found to have an increase in *TP53* mutations, albeit only with sample sizes of *n*=5 [43]. Thus, there is abundant indirect evidence suggesting that these genes deserve further investigation as possible candidates for addiction in the absence of functional *TP53*. More generally, we anticipate that improved tests for differential essentiality, such as the ones introduced here, combined with rapidly growing CRISPR-screen data sets, will enable the detection of many new cases of genotype-dependent differences in essentiality, including both synthetic lethality and non-oncogene addiction.

## Conclusions

In this paper, we have introduced the ACE modeling framework for CRISPR/Cas9 screens. We show that ACE is competitive with the best available methods in predicting essentiality, and in addition, supports a powerful new test for differential essentiality. In addition, we show using real data how this test for differential essentiality can uncover unique genetic dependencies that might otherwise be overlooked, focusing in particular on the case of a *TP53*-linked dependence on RNA methyltransferases in NSCLC lung samples. The ACE software is freely available for use by the scientific community.

## Methods

### Initial infection

The initial abundance *n*_*sg*_ of each sgRNA *g* within sample *s* is assumed to be given by a Poisson distribution with mean *μ*_1_=*c*_*s*_*m*_*g*_, where *c*_*s*_ is the total number of cells infected in the screen and *m*_*g*_ is the relative frequency of *g* in the master library:

1 $$ P\left(n_{sg} | c_{s},m_{g}\right) = \text{Pois}\left(n_{sg} | \mu_{1} = c_{s} m_{g}\right).   $$

The value *m*_*g*_ for each sgRNA *g* is estimated as the fraction of sequence reads corresponding to *g* in the sequence data for the master library. If multiple replicates are available, an average is taken. The parameter *c*_*s*_ is either provided by the user or a default value of 1000 is assumed.

### Initial read counts

The sgRNA sequencing counts *x*_*sg*_ from the initial infected population *n*_*sg*_ are assumed to follow a Poisson distribution with mean *μ*_2_=*γ*_*s*_*n*_*sg*_, where *γ*_*s*_ is a scaling parameter that captures the relationship between sequencing depth and the number of cells infected:

2 $$ P\left(x_{sg} | \gamma_{s}, n_{sg}\right) = \text{Pois} \left(x_{sg} | \mu_{2} = \gamma_{s} n_{sg}\right).   $$

### Final read counts

Following several rounds of cell division, the final abundance of each sgRNA *g* is assumed to be *d*_*sg*_=*n*_*sg*_(1−*ε*_*g*_*ϕ*_*G*_), where *ϕ*_*G*_ is the essentiality of the gene *G* that is targeted by *g*, and *ε*_*g*_ is the efficiency of *g*. This process is assumed to be deterministic; we assume that the number of infected cells is sufficiently large and sufficiently many cell divisions have occurred that stochastic effects can be ignored, and uncertainty in the value of *d*_*sg*_ can be absorbed by the distributions for *n*_*sg*_ and *y*_*sg*_. The final read counts *y*_*sg*_ are then assumed to follow a Poisson distribution with mean *μ*_3_=*γs*′*d*_*sg*_, where *γs*′ is the final read count scaling factor:

3 $$\begin{array}{*{20}l} P\left(y_{sg} | d_{sg}, \gamma\rq_{s}, \epsilon_{g}\phi_{G}\right) &= \text{Pois} \left(y_{sg} | \mu_{3} = \gamma\rq_{s} d_{sg}\right)  \\ &= \text{Pois} \left(y_{sg} | \mu_{3}= \gamma\rq_{s} n_{sg} (1-\epsilon_{g}\phi_{G})\right).  \end{array} $$

The scaling factor *γs*′ not only captures the relationship between sequencing depth and the number of cells that remain after cell growth, but also can accommodate sample-specific differences in the growth rate of all cells (independent of sgRNA).

### Guide-specific effects

The sgRNA-specific efficiency *ε*_*g*_ is derived from a general feature vector by logistic regression:

4 $$ \epsilon_{g} = \frac{\exp(\omega \cdot F_{g})}{1+\exp(\omega \cdot F_{g})},   $$

where *F*_*g*_ is a feature vector and *ω* is a corresponding vector of real-valued coefficients. In this paper, we used a ten-dimensional vector *F*_*g*_ whose elements are indicators (via “one-hot” encoding) for the decile of GC content of each sgRNA’s template guide. The corresponding 10-dimensional weight vector *ω* was treated as a free parameter and estimated by maximum likelihood. In this way, we effectively estimate a separate efficiency value for each GC decile, based on pooled information from all guides within that decile. As noted in the text, users may instead wish to use richer feature vectors such as those described in other recent studies [26, 44–48].

### Estimation of sample scaling parameters

Following the “median-of-ratios” approach used by DESeq2 [5], the sample scaling parameters *γ*_*s*_ and *γs*′ are not treated as free parameters in the maximization of the likelihood, but instead are set in a preprocessing step based on the raw read counts *x*_*sg*_ and *y*_*sg*_ for negative controls. Specifically, the scaling parameter is set equal to the median of the ratios of each sgRNA’s read count to a guide-specific reference value, which in this case is the expected number of infected cells, *c*_*s*_*m*_*g*_:

5 $$ \gamma_{s} = \underset{g\in \mathrm{G}_{\text{neg}}}{\text{median}} \left(\frac{x_{sg}}{c_{s} m_{g}}\right), \qquad \gamma\rq_{s} = \underset{g\in \mathrm{G}_{\text{neg}}}{\text{median}} \left(\frac{y_{sg}}{c_{s} m_{g}}\right),   $$

where *g*∈*G*_*neg*_ indicates all sgRNAs targeting genes in the designated set of negative controls. If no master library is available, the initial abundance of sgRNA $\bar g$ is approximated from the initial read counts as the average across samples:

6 $$ m_{\bar g} \approx \frac{1}{S} \sum_{s=1}^{S}{\frac{x_{s\bar g}}{\sum_{g}{x_{sg}}}},  $$

where *S* is the total number of samples. The user may choose to use the median ratio across all genes rather than only the negative controls, if desired. This heuristic method of estimation may prove insufficient for some experimental cases, such as misclassified negative controls or strong, sample-specific multipliers of essentiality affecting all genes in experiments with few samples, though we found these estimates satisfactory for the analyses and benchmarking presented in this work.

### Full model likelihood

Substituting from Eqs. 1 to 3, the full likelihood function can be expressed as:

7 $$ \begin{aligned} L(\phi_., \omega_.;\, x_{..}, y_{..}, m_., c_., \gamma_., \gamma\rq_.) &= \prod_{G} \prod_{g\in G} \prod_{s} \sum_{n_{sg} = 0}^{c_{s}} \text{Pois} \left(n_{sg} | c_{s} m_{g}\right) \text{Pois} \left(x_{sg} |\gamma_{s} n_{sg} \right) \\ & \qquad\qquad\qquad\quad \times \text{Pois}\left(y_{sg} |\gamma\rq_{s}n_{sg} (1-\epsilon_{g} \phi_{G}) \right)  \end{aligned}  $$

where *ε*_*g*_ is implicitly a function of *ω* as defined by Eq. 4. Here and below we use “dot” notation to indicate sets of all relevant indices (e.g., *x*_.._ = {*x*_*sg*_ | for all samples*s* and sgRNAs *g*}).

The likelihood function must sum over the number of infected cells *n*_*sg*_, which cannot be directly observed. This summation step can be accelerated by an approximation that bundles summands together, for example, into groups of 10 cells.

When *ω* is held fixed, the full likelihood decomposes by gene, such that each *ϕ*_*G*_ can be estimated separately, and similarly, when the *ϕ*_*G*_ values are held fixed, the likelihood decomposes by the efficiency features *f*, owing to our “one-hot” design for the feature vector. Thus, we estimate each *ϕ*_*G*_ and component of *ω*_*f*_ iteratively, to enable parallelization by gene and feature category. Specifically, we repeatedly carry out the following optimizations until convergence (for each gene *G* and feature *f*, respectively):

8 $$ \begin{aligned} \widehat{\phi}_{G} = \underset{\phi_{G}}{\text{argmax}}\big\lbrace L(\phi_{G}, \widehat{\phi}_{\neg G}, \widehat{\omega};\, x_{..}, y_{..}, \theta) \big\rbrace \\ \widehat{\omega}_{f} = \underset{\omega_{f}}{\text{argmax}}\big\lbrace L(\widehat{\phi}_., \omega_{f}, \widehat{\omega}_{\neg f};\, x_{..},y_{..}, \theta) \big\rbrace \end{aligned}  $$

where *ϕ*_¬*G*_ denotes the set {*ϕ*_*g*_ | *g*≠*G*},*ω*_¬*f*_ denotes the set $\phantom {\dot {i}\!}\{\omega _{f'} \;|\; f' \ne f\}$, and *θ* denotes the remaining parameter set. We initialize *ω* to treat all sgRNA as 100% effective.

Finally, to evaluate whether an estimated value of $ \widehat {\phi }_{G} $ is significantly different from zero, we compare the component of the maximized likelihood function for that gene to the corresponding component when *ϕ*_*G*_ is held fixed at zero. Specifically, we compute a test statistic *T* as,

9 $$ T = \text{ln} \frac{L(\widehat{\phi}_{G})}{L(\phi_{G} = 0)},   $$

where *L*(*ϕ*_*G*_) represents the component of the likelihood corresponding to gene *G*. We compute an empirical *p*-value for *T* based on the equivalent test statistics for the designated negative control genes.

### Adaptation for alternative experimental designs

The likelihood function can easily be adapted to settings in which data is unavailable for either the master library or the initial sgRNA abundances. In the case where the master library information is missing, we simply use a uniform prior for values of $n_{sg}, P(n_{sg}|c_{s}) = \frac 1{c_{s}}$. When the initial infected sgRNA abundances are unavailable, we remove the corresponding portion of the likelihood function, effectively treating *x*_.._ as missing data:

10 $$ \begin{aligned} L(\phi_., \omega_.;\,x_{..}=\emptyset, y_{..}, m_., c_., \gamma_., \gamma\rq_.) &= \prod_{G} \prod_{g\in G} \prod_{s} \sum_{n_{sg} = 0}^{c_{s}} \text{Pois} \left(n_{sg} | c_{s} m_{g}\right) \\ & \qquad\qquad\qquad\quad\times \text{Pois}\left(y_{sg} |\gamma\rq_{s}n_{sg} (1-\epsilon_{g} \phi_{G}) \right) \end{aligned}  $$

### ACE test for differential essentiality

To identify genes with significant differences in essentiality between a “test” set of samples, *S*_*t*_, and a “control” set, *S*_*c*_, we employ the following likelihood ratio test. Let $L(\widehat \phi _{G})$ represent the component of the likelihood function relevant to gene *G* that has been maximized in the standard way, with one version of the *ϕ*_*G*_ parameter shared across both *S*_*t*_ and *S*_*c*_. By contrast, let $L(\widehat \phi _{G}^{t}) L(\widehat \phi _{G}^{c})$ represent the corresponding component of the likelihood optimized such that separate versions of the essentiality parameter, $\phi _{G}^{t}$ and $\phi _{G}^{c}$, are used for samples *S*_*t*_ and *S*_*c*_, respectively. The likelihood ratio test statistic *T*^′^ is then given by,

11 $$ T' = \ln \frac{L(\widehat\phi_{G}^{t})L(\widehat\phi_{G}^{c})}{L(\widehat\phi_{G})}.  $$

For this test, all samples are used to calculate the sample-specific parameters *γ*_*s*_ and *γs*′, and the sgRNA-specific parameter *ε*_*g*_; only $\phi _{G}^{t}$ and $\phi _{G}^{c}$ are re-estimated to compute *T*^′^. As with the test for absolute essentiality, an empirical *p*-value is computed for *T*^′^ based on the distribution of values computed for the negative controls. In some analyses, we performed a one-sided test by further requiring that $\widehat \phi _{G}^{t} > \widehat \phi _{G}^{c}$ and otherwise setting *T*^′^ to 0.

### Essential and nonessential controls

The set of essential genes used for validation was defined as those genes that were included in at least two of three previously published sets of essential genes [1–3]. The set of nonessential genes was obtained from 604 total RNA-seq datasets for 128 cell lines, in vitro differentiated cells, primary cells, and tissues accessed from the Encyclopedia of DNA Elements (ENCODE) data portal on August 29, 2019 (see Additional file 2 for accession info of all cell lines used). For the nonessential set, we chose 594 genes expressed at ≤1 TPM in all cell lines.

### Data from cancer dependency map (DepMap)

For our analysis of real data from genome-wide CRISPR knockout screens, we used two large databases available from the Cancer Dependency Map (DepMap) Consortium: Project Achilles, produced by the Broad Institute [9], and Project Score, produced by the Sanger Institute [49, 50]. The Achilles Project dataset uses the Avana library and includes 1374 publicly available screens in 625 unique cell lines at the time of writing. Only the pre-infection master library and the post-depletion sgRNAs were sequenced, with one to four replicates per cell line available. Following the Achilles DepMap convention [51], we assume a gene has lost function (indicated as *Δ*) in all CCLE cell lines annotated as containing one or more of the following: single nucleotide polymorphisms (SNPs), deletions, or insertions in the start codon; deletions or insertions that introduce a frameshift mutation; mutations that introduce a premature stop codon or a de novo frameshifted start codon; or mutations that disrupt a splice site [32, 33].

For benchmarking with differential essential genes, we took three different approaches. For the first, we relied upon gene expression from the previously described ENCODE RNA-seq data sets to nominate our differentially essential gene set. The median expression of each gene was evaluated separately across the 206 unique lung samples and the 24 unique liver samples, with a total of 42 liver replicates and 42 lung replicates subsampled for data in the final analysis. 494 genes with a median expression difference ≥1.5 TPM were chosen to represent differentially essential genes (see Additional file 1: Fig. S6). Genes with high levels of expression in both tissue panels (>5 TPM) were excluded in an effort to isolate genes essential to only one tissue type. No genes were “perfectly” differential, with no expression in all samples of one panel, and detectable expression in all samples of the other. Consequently, by the imperfect proxy of gene expression, there is overlap in the by-sample essentiality of our “differentially essential” gene sets.

The uniformly active gene set is composed of 698 genes with expression levels >5 TPM in all lung- and liver-derived samples evaluated, according to previously published gene expression data for these cell lines. The uniformly silent gene set contains 594 genes with expression <1 TPM across all samples; 42 of these genes were randomly selected and provided as annotated negative controls to all essentiality inference methods save CERES, for which by-sample precomputed scores were used (see below).

For our second benchmarking analysis in real data, we focused on two known differentially essential genes, *CDKN1A* and *MDM2*, and randomly selected the various numbers of samples compared. To retain a similar genetic background between sample panels, samples were compared from the same tissue type and lineage, specifically cell lines originating from lung non-small cell lung cancer (NSCLC) adenocarcinoma. The Achilles Project dataset includes 220 replicates of 92 unique samples of these cell lines.

For our third benchmarking analysis in real data, we randomly selected three samples for each panel from cell lines across five tissues of origin. In this case, no replicates were used. We chose 19 differentially essential genes and their associated genetic backgrounds according to the RNAi-based findings of Project Drive [38], assuming differential essentiality to be universal across all cell lines. We subsampled 500 negative and positive controls from the previously mentioned control gene sets and contrasted them with the rankings of the differentially essential genes according to their test statistics. In addition, we randomly selected 50 examples from each control set and provided them as annotated controls to all essentiality inference methods save CERES, for which by-sample precomputed scores were used (see below).

The Project Score dataset has assayed 323 human cancer cell lines using the Human CRISPR Library [19]. For our test of RNA methyltransferase differential essentiality, we compared the essentiality signals of the six available screens in cell lines having wildtype *TP53* (cell lines LU998 and H322M) with those from six screens from two of the cell lines with non-functional *TP53* (LXF289 and H1650).

### empiriCRISPR simulation of CRISPR knockout screens

We designed the empiriCRISPR screen simulator to mimic the empirical properties of a template data set as closely as possible, while minimizing shared properties with our ACE inference model. empiriCRISPR simulates fractional sgRNA abundance sampled from a template screen and uses a series of gamma distributions to generate initial and final screen counts, $\widehat {x}_.. $ and $\widehat {y}_{..}$, as follows:

12 $$ \widehat{\mu}_{..} = \text{Gamma}(\alpha_{.},\beta{.}), \qquad \alpha_{.}=\frac{\sigma_{m}}{m_{.}}, \qquad \beta_{.}=\frac{m^{2}_{.}}{\sigma_{m}}  $$

13 $$ \widehat{x}_{..} = X_. \text{Gamma}(\alpha'_{..}, \beta'_{..}), \qquad \alpha'_{..} = \frac{\sigma_{x}}{\widehat{\mu}_{..}}, \qquad \beta'_{..} = \frac{\widehat{\mu}^{2}_{..}}{\sigma_{x}}  $$

14 $$ \widehat{y}_{..} = Y_. \text{Gamma}(\alpha^{\prime\prime}_{..},\beta^{\prime\prime}_{..}), \quad \alpha^{\prime\prime}_{..} = \frac{\sigma_{y}}{[1-\epsilon_. \phi_.]\widehat{\mu}_{..}}, \quad \beta^{\prime\prime}_{..} = \frac{([1-\epsilon_. \phi_.]\widehat{\mu}_{..})^{2}}{\sigma_{y}}  $$

where *X*_._ and *Y*_._ are each sample’s target total read counts in the initial and final sequencing and *m*_._ is the abundance of each guide in the simulation’s template master library. The parameters *ε*_._ and *ϕ*_._ are the sgRNA efficiency and gene essentiality chosen for the simulation. empiriCRISPR simulates each sgRNA independently using *α* and *β* parameters derived from empirical estimates of variation from infection, initial, and final sequencing (*σ*_*m*_,*σ*_*x*_, and *σ*_*y*_, respectively), fit across replicates of the provided template screen. empiriCRISPR calculates the gamma distribution parameters by minimizing the adjusted mean squared error of the summary statistics of simulated data with the median of 5 summary statistics of the template screens, shown in Additional file 1: Fig. S3. The actual variation of sgRNA abundances *within* a sample is wholly dependent on the number of sgRNA and template master library chosen.

This implementation of empiriCRISPR is independent of the probabilistic model and parameter inference used in ACE, save for the general structure of the hierarchical model based on CRISPR screens. While the read counts in empiriCRISPR are derived from flexible gamma distributions designed to capture as much of the biological variation of template CRISPR screens as possible, ACE makes no attempt to infer specific variation parameters. CRISPR knockout screens from [16] were used as the empiriCRISPR template, and initial master library abundances were selected by sampling the template master sgRNA library. Unless otherwise noted, simulations used 300 genes per essentiality value, four sgRNA per gene, and three samples, and assumed a constant essentiality *ϕ*_*G*_ across all sgRNA and samples for gene *G*.

### Software package

ACE is implemented as an R package (R 3.5.0, [52]) that uses *data.table* [53] and *R6* [54]. Optimization is performed using R’s *optim* function with the “Brent” method. Both the ACE and empiriCRISPR github repositories can be found at https://github.com/CshlSiepelLab. ACE can be run across all genes in parallel. Optimization takes several seconds per gene (see Additional file 1: Fig. S19). The run-time of the software scales approximately linearly with the number of samples included.

### Estimation of essentiality from mean log-fold change

The “average fold change” (“AFC”) score for each gene *G* was calculated as follows:

15 $$ \text{AFC} = 1 - \prod_{g=1}^{g\in G} \prod_{s=1}^{S} \left(\lambda_{s} \tau_{neg} \frac{(0.5 + y_{sg}) }{(0.5 + x_{sg})}\right)^{\frac{1}{SG}}   $$

16 $$ \tau_{neg}^{-1} = \prod_{g=1}^{g\in G_{neg}} \prod_{s=1}^{S} \left(\lambda_{s} \frac{(0.5 + y_{sg}) }{(0.5 + x_{sg})}\right)^{\frac{1}{SG_{neg}}},  $$

where a pseudocount of 0.5 was added to both initial (*x*_*sg*_) and final (*y*_*sg*_) read counts to prevent undefined values. Each sample was normalized by *λ*_*s*_ to bring the total read count of each sample to a total of 10 million reads, and final fold changes were adjusted according to *τ*_*neg*_, the mean fold change in all negative controls *G*_*neg*_. Consistent with our ACE notation, an AFC value of zero has no impact on cell growth or proliferation.

### Estimation of essentiality using BAGEL

We applied the BAGEL (Bayesian Analysis of Gene Essentiality) method [1] using software version 0.91 (last modified 09/2015). Simulated data was analyzed using 4% of genes as positive and negative controls, with simulated essentiality scores of 0.99 (99% depletion) and 0.05 (5% depletion) respectively. Differential essentiality was calculated as the difference of Bayes Factors between separate analysis of test and control sample panels, as performed in [1]. For the analysis of real data, we used all genes identified as essential or nonessential in our earlier analysis.

### Estimation of essentiality using JACKS

We applied the JACKS (Joint Analysis of CRISPR/Cas9 Knock-out Screens) method [8] version 0.2 (downloaded 11/2019). Differential essentiality was evaluated by running JACKS separately for the test and control sample panels, using the built-in hierarchical prior option and the same negative control genes provided to other methods for both real and simulated data. The test statistic for differential essentiality was indicated by the ratio in sample-specific *p*-value in the case of only one sample type (as in simulated data in Figs. 2 and 3), and by a Student’s *t*-test in the case of panels containing multiple sample types (as in Fig. 4E). While JACKS also reports gene-knockout effects, they are not normalized by sample [49], and the *p*-values used reflect the sample-dependent null distributions inferred by JACKS. For Fig. 4E and Additional file 1: Fig. S9, a one-sided *t*-test of the appropriate direction was separately performed for the statistics of *MDM2* and *CDKN1A*, as *CDKN1A* is a tumor suppressor with a “negative” gene essentiality. In the case of bulk gene analysis in Additional file 1: Figs. S7 and S8, a two-sided *t*-test was used.

### Estimation of essentiality using CRISPhieRmix

We used the CRISPhieRmix software version 0.1.0, using 100 points of integration (parameter “nMesh”) and the “BIMODAL” testing option. While the software is optimized for use in large pooled CRISPRi and CRISPRa screens, it may also be applied to knockout screens as demonstrated by the authors in [23]. Differential essentiality was estimated by separately running CRISPhieRmix on test and control sample panels, and the test statistic determined by the ratio of the gene essentiality false discovery rates between the two panels.

### Estimation of essentiality using CERES

Previously published CERES-based *p*-values were obtained for each Achilles DepMap sample used in the methods comparison. As in ref. [9], the CERES differential essentiality test statistic was calculated using a Student’s *t*-test to compare the by-sample log fold change effect sizes of the test and control sample panels. For Fig. 4E and Additional file 1: Fig. S9, a one-sided *t*-test of the appropriate direction was separately performed for the statistics of *MDM2* and *CDKN1A*, as *CDKN1A* is a tumor suppressor with a “negative” gene essentiality. In the case of bulk gene analysis in Additional file 1: Figs. S7 and S8, a two-sided *t*-test was used.

## Supplementary Information

**Additional file 1** Supplemental figures and analysis.

**Additional file 2** Accession numbers of RNAseq assays used in nonessential and tissue-specific gene set curation.

**Additional file 3** Review history.

## Data Availability

The software is freely available at https://github.com/CshlSiepelLab/ACE under the MIT license (archived with Zenodo at 10.5281/zenodo.5493071). The set of nonessential genes was obtained from 604 total RNA-seq datasets for 128 cell lines, in vitro differentiated cells, primary cells, and tissues accessed from the Encyclopedia of DNA Elements (ENCODE) data portal on August 29, 2019 (see Additional file 2 for accession info of all cell lines used). The CRISPR screens used as the template for our simulation study are available from [16], specifically the raw read counts for the genome-wide CRISPR screens in cell lines DLD1 and HCT116 in Supplemental Tables 1 and 3. Pan-essential gene sets were derived from the 361 essential genes from ’‘Core Essential Gene Set 1” (from [55]), from the 1580 essential genes from [2], and from the 684 ’‘Core Essential Gene Set 2” (from [3], Supplemental Table 2). The raw read counts from the genome-wide CRISPR screens from the DepMap Project Achilles were downloaded from DepMap Public 19Q3 release from the DepMap Data Portal ([51] on 10/12/19). The raw read counts from the DepMap Project Score were downloaded on 1/14/19 from the precursor site to the Project Score database (https://score.depmap.sanger.ac.uk/downloads).

## References

[1] Hart T, Moffat J (2016). BAGEL: a computational framework for identifying essential genes from pooled library screens. BMC Bioinformatics.

[2] Hart T, Chandrashekhar M, Aregger M, Steinhart Z, Brown KR, MacLeod G, Mis M, Zimmermann M, Fradet-Turcotte A, Sun S, Mero P, Dirks P, Sidhu S, Roth FP, Rissland OS, Durocher D, Angers S, Moffat J (2015). High-resolution CRISPR screens reveal fitness genes and genotype-specific cancer liabilities. Cell.

[3] Hart T, Tong AHY, Chan K, Van Leeuwen J, Seetharaman A, Aregger M, Chandrashekhar M, Hustedt N, Seth S, Noonan A, Habsid A, Sizova O, Nedyalkova L, Climie R, Tworzyanski L, Lawson K, Sartori MA, Alibeh S, Tieu D, Masud S, Mero P, Weiss A, Brown KR, Usaj M, Billmann M, Rahman M, Constanzo M, Myers CL, Andrews BJ, Boone C, Durocher D, Moffat J (2017). Evaluation and design of genome-wide CRISPR/SpCas9 knockout screens. G3:Genes|Genomes|Genetics.

[4] Li W, Xu H, Xiao T, Cong L, Love MI, Zhang F, Irizarry R. a., Liu JS, Brown M, Liu XS (2014). MAGeCK enables robust identification of essential genes from genome-scale CRISPR/Cas9 knockout screens. Genome Biol.

[5] Love MI, Huber W, Anders S (2014). Moderated estimation of fold change and dispersion for RNA-seq data with DESeq2. Genome Biol.

[6] Dai Z, Sheridan JM, Gearing LJ, Moore DL, Su S, Wormald S, Wilcox S, O’Connor L, Dickins RA, Blewitt ME, Ritchie ME (2014). edgeR: a versatile tool for the analysis of shRNA-seq and CRISPR-Cas9 genetic screens. F1000Research.

[7] Noh J, Chen B. sgRSEA: Enrichment Analysis of CRISPR/Cas9 Knockout Screen Data. 2015. https://cran.r-project.org/src/contrib/Archive/sgRSEA/.

[8] Allen F, Behan F, Khodak A, Iorio F, Yusa K, Garnett M, Parts L (2019). JACKS: joint analysis of CRISPR/Cas9 knockout screens. Genome Res.

[9] Meyers RM, Bryan JG, McFarland JM, Weir BA, Sizemore AE, Xu H, Dharia NV, Montgomery PG, Cowley GS, Pantel S, Goodale A, Lee Y, Ali LD, Jiang G, Lubonja R, Harrington WF, Strickland M, Wu T, Hawes DC, Zhivich VA, Wyatt MR, Kalani Z, Chang JJ, Okamoto M, Stegmaier K, Golub TR, Boehm JS, Vazquez F, Root DE, Hahn WC, Tsherniak A (2017). Computational correction of copy number effect improves specificity of CRISPR-Cas9 essentiality screens in cancer cells. Nat Genet.

[10] Yu J, Silva J, Califano A (2015). ScreenBEAM: A novel meta-analysis algorithm for functional genomics screens via Bayesian hierarchical modeling. Bioinformatics.

[11] Morgens DW, Deans RM, Li A, Bassik MC (2016). Systematic comparison of CRISPR/Cas9 and RNAi screens for essential genes SUPPLEMENT. Nat Biotechnol.

[12] Evers B, Jastrzebski K, Heijmans JPM, Grernrum W, Beijersbergen RL, Bernards R (2016). CRISPR knockout screening outperforms shRNA and CRISPRi in identifying essential genes. Nat Biotechnol.

[13] Inoue F, Kircher M, Martin B, Cooper GM, Witten DM, McManus MT, Ahituv N, Shendure J (2017). A systematic comparison reveals substantial differences in chromosomal versus episomal encoding of enhancer activity. Genome Res.

[14] Wang T, Birsoy K, Hughes NW, Krupczak KM, Post Y, Wei JJ, Lander ES, Sabatini DM (2015). Identification and characterization of essential genes in the human genome. Science.

[15] Wang T, Yu H, Hughes NW, Chen WW, Lander ES, Sabatini DM (2017). Gene essentiality profiling reveals gene networks and synthetic lethal interactions with oncogenic Ras. Cell.

[16] Martin TD, Cook DR, Choi MY, Li MZ, Haigis KM, Elledge SJ (2017). A role for mitochondrial translation in promotion of viability in K-Ras mutant cells. Cell Rep.

[17] Tarumoto Y, Lu B, Somerville TDD, Huang YH, Milazzo JP, Wu XS, Klingbeil O, El Demerdash O, Shi J, Vakoc CR (2018). LKB1, salt-inducible kinases, and MEF2C are linked dependencies in acute myeloid leukemia. Mol Cell.

[18] Aguirre AJ, Meyers RM, Weir BA, Vazquez F, Zhang CZ, Ben-David U, Cook A, Ha G, Harrington WF, Doshi MB, Kost-Alimova M, Gill S, Xu H, Ali LD, Jiang G, Pantel S, Lee Y, Goodale A, Cherniack AD, Oh C, Kryukov G, Cowley GS, Garraway LA, Stegmaier K, Roberts CW, Golub TR, Meyerson M, Root DE, Tsherniak A, Hahn WC (2016). Genomic copy number dictates a gene-independent cell response to CRISPR/Cas9 targeting. Cancer Discov.

[19] Tzelepis K, Koike-Yusa H, De Braekeleer E, Li Y, Metzakopian E, Dovey O, Mupo A, Grinkevich V, Li M, Mazan M, Gozdecka M, Ohnishi S, Cooper J, Patel M, McKerrell T, Chen B, Domingues A, Gallipoli P, Teichmann S, Ponstingl H, McDermott U, Saez-Rodriguez J, Huntly BP, Iorio F, Pina C, Vassiliou G, Yusa K (2016). A CRISPR dropout screen identifies genetic vulnerabilities and therapeutic targets in acute myeloid leukemia. Cell Rep.

[20] Nagel R, Semenova EA, Berns A (2016). Drugging the addict: non-oncogene addiction as a target for cancer therapy. EMBO Rep.

[21] Cully M (2019). Chemical inhibitors make their RNA epigenetic mark. Nat Rev Drug Discov.

[22] Jia G, Wang X, Xiao G (2017). A permutation-based non-parametric analysis of CRISPR screen data. BMC Genomics.

[23] Daley TP, Lin Z, Lin X, Liu Y, Wong WH, Qi LS (2018). CRISPhieRmix: A hierarchical mixture model for CRISPR pooled screens. Genome Biol.

[24] Spahn PN, Bath T, Weiss RJ, Kim J, Esko JD, Lewis NE, Harismendy O. PinAPL-Py: A comprehensive web-application for the analysis of CRISPR/Cas9 screens. Sci Rep. 2017; 7(1). 10.1038/s41598-017-16193-9. 10.1038/s41598-017-16193-9 PMC5696473 29158538

[25] Sullivan K. The Cancer Dependency Map Consortium. 2021. https://depmap.org/portal/static/img/dmc/depmap_consortium.pdf.

[26] Doench JG, Fusi N, Sullender M, Hegde M, Vaimberg EW, Donovan KF, Smith I, Tothova Z, Wilen C, Orchard R, Virgin HW, Listgarten J, Root DE (2016). Optimized sgRNA design to maximize activity and minimize off-target effects of CRISPR-Cas9. Nat Biotechnol.

[27] Pinello L, Canver MC, Hoban MD, Orkin SH, Kohn DB, Bauer DE, Yuan GC (2016). Analyzing CRISPR genome-editing experiments with CRISPResso. Nat Biotechnol.

[28] Jinek M, Jiang F, Taylor DW, Sternberg SH, Kaya E, Ma E, Anders C, Hauer M, Zhou K, Lin S, Kaplan M, Iavarone AT, Charpentier E, Nogales E, Doudna J. a. (2014). Structures of Cas9 endonucleases reveal RNA-mediated conformational activation. Science (New York, N.Y.).

[29] Unckless RL, Clark AG, Messer PW (2017). Evolution of resistance against CRISPR/Cas9 gene drive. Genetics.

[30] Yau EH, Kummetha IR, Lichinchi G, Tang R, Zhang Y, Rana TM (2017). Genome-wide CRISPR screen for essential cell growth mediators in mutant KRAS colorectal cancers. Cancer Res.

[31] Scully KH, Jacobson EM, Jepsen K, Lunyak V, Viadiu H, Carriere C, Rose DW, Hooshmand F, Aggarwal AK, Rosenfeld MG (2000). Allosteric effects of Pit-1 DNA sites on long-term repression in cell type specification. Science.

[32] Barretina J, Caponigro G, Stransky N, Venkatesan K, Margolin AA, Kim S, Wilson CJ, Lehár J, Kryukov GV, Sonkin D, Reddy A, Liu M, Murray L, Berger MF, Monahan JE, Morais P, Meltzer J, Korejwa A, Jané-Valbuena J, Mapa FA, Thibault J, Bric-Furlong E, Raman P, Shipway A, Engels IH, Cheng J, Yu GK, Yu J, Aspesi P, De Silva M, Jagtap K, Jones MD, Wang L, Hatton C, Palescandolo E, Gupta S, Mahan S, Sougnez C, Onofrio RC, Liefeld T, MacConaill L, Winckler W, Reich M, Li N, Mesirov JP, Gabriel SB, Getz G, Ardlie K, Chan V, Myer VE, Weber BL, Porter J, Warmuth M, Finan P, Harris JL, Meyerson M, Golub TR, Morrissey MP, Sellers WR, Schlegel R, Garraway LA (2012). The Cancer Cell Line Encyclopedia enables predictive modelling of anticancer drug sensitivity. Nature.

[33] Ghandi M, Huang FW, Jané-Valbuena J, Kryukov GV, Lo CC, McDonald ER, Barretina J, Gelfand ET, Bielski CM, Li H, Hu K, Andreev-Drakhlin AY, Kim J, Hess JM, Haas BJ, Aguet F, Weir BA, Rothberg MV, Paolella BR, Lawrence MS, Akbani R, Lu Y, Tiv HL, Gokhale PC, de Weck A, Mansour AA, Oh C, Shih J, Hadi K, Rosen Y, Bistline J, Venkatesan K, Reddy A, Sonkin D, Liu M, Lehar J, Korn JM, Porter DA, Jones MD, Golji J, Caponigro G, Taylor JE, Dunning CM, Creech AL, Warren AC, McFarland JM, Zamanighomi M, Kauffmann A, Stransky N, Imielinski M, Maruvka YE, Cherniack AD, Tsherniak A, Vazquez F, Jaffe JD, Lane AA, Weinstock DM, Johannessen CM, Morrissey MP, Stegmeier F, Schlegel R, Hahn WC, Getz G, Mills GB, Boehm JS, Golub TR, Garraway LA, Sellers WR (2019). Next-generation characterization of the Cancer Cell Line Encyclopedia. Nature.

[34] Frum RA, Grossman SR (2014). Mechanisms of mutant p53 stabilization in cancer. Sub-Cellular Biochem.

[35] Lavin MF, Gueven N (2006). The complexity of p53 stabilization and activation. Cell Death Differ.

[36] Mueller S, Engleitner T, Maresch R, Zukowska M, Lange S, Kaltenbacher T, Konukiewitz B, Öllinger R, Zwiebel M, Strong A, Yen HY, Banerjee R, Louzada S, Fu B, Seidler B, Götzfried J, Schuck K, Hassan Z, Arbeiter A, Schönhuber N, Klein S, Veltkamp C, Friedrich M, Rad L, Barenboim M, Ziegenhain C, Hess J, Dovey OM, Eser S, Parekh S, Constantino-Casas F, De La Rosa J, Sierra MI, Fraga M, Mayerle J, Klöppel G, Cadiñanos J, Liu P, Vassiliou G, Weichert W, Steiger K, Enard W, Schmid RM, Yang F, Unger K, Schneider G, Varela I, Bradley A, Saur D, Rad R (2018). Evolutionary routes and KRAS dosage define pancreatic cancer phenotypes. Nature.

[37] Sanchez-Vega F, Mina M, Armenia J, Chatila WK, Luna A, La KC, Dimitriadoy S, Liu DL, Kantheti HS, Saghafinia S, Chakravarty D, Daian F, Gao Q, Bailey MH, Liang WW, Foltz SM, Shmulevich I, Ding L, Heins Z, Ochoa A, Gross B, Gao J, Zhang H, Kundra R, Kandoth C, Bahceci I, Dervishi L, Dogrusoz U, Zhou W, Shen H, Laird PW, Way GP, Greene CS, Liang H, Xiao Y, Wang C, Iavarone A, Berger AH, Bivona TG, Lazar AJ, Hammer GD, Giordano T, Kwong LN, McArthur G, Huang C, Tward AD, Frederick MJ, McCormick F, Meyerson M, Caesar-Johnson SJ, Demchok JA, Felau I, Kasapi M, Ferguson ML, Hutter CM, Sofia HJ, Tarnuzzer R, Wang Z, Yang L, Zenklusen JC, Zhang JJ, Chudamani S, Liu J, Lolla L, Naresh R, Pihl T, Sun Q, Wan Y, Wu Y, Cho J, DeFreitas T, Frazer S, Gehlenborg N, Getz G, Heiman DI, Kim J, Lawrence MS, Lin P, Meier S, Noble MS, Saksena G, Voet D, Zhang H, Bernard B, Chambwe N, Dhankani V, Knijnenburg T, Kramer R, Leinonen K, Liu Y, Miller M, Reynolds S, Shmulevich I, Thorsson V, Zhang W, Akbani R, Broom BM, Hegde AM, Ju Z, Kanchi RS, Korkut A, Li J, Liang H, Ling S, Liu W, Lu Y, Mills GB, Ng KS, Rao A, Ryan M, Wang J, Weinstein JN, Zhang J, Abeshouse A, Armenia J, Chakravarty D, Chatila WK, de Bruijn I, Gao J, Gross BE, Heins ZJ, Kundra R, La K, Ladanyi M, Luna A, Nissan MG, Ochoa A, Phillips SM, Reznik E, Sanchez-Vega F, Sander C, Schultz N, Sheridan R, Sumer SO, Sun Y, Taylor BS, Wang J, Zhang H, Anur P, Peto M, Spellman P, Benz C, Stuart JM, Wong CK, Yau C, Hayes DN, Parker JS, Wilkerson MD, Ally A, Balasundaram M, Bowlby R, Brooks D, Carlsen R, Chuah E, Dhalla N, Holt R, Jones SJM, Kasaian K, Lee D, Ma Y, Marra MA, Mayo M, Moore RA, Mungall AJ, Mungall K, Robertson AG, Sadeghi S, Schein JE, Sipahimalani P, Tam A, Thiessen N, Tse K, Wong T, Berger AC, Beroukhim R, Cherniack AD, Cibulskis C, Gabriel SB, Gao GF, Ha G, Meyerson M, Schumacher SE, Shih J, Kucherlapati MH, Kucherlapati RS, Baylin S, Cope L, Danilova L, Bootwalla MS, Lai PH, Maglinte DT, Van Den Berg DJ, Weisenberger DJ, Auman JT, Balu S, Bodenheimer T, Fan C, Hoadley KA, Hoyle AP, Jefferys SR, Jones CD, Meng S, Mieczkowski PA, Mose LE, Perou AH, Perou CM, Roach J, Shi Y, Simons JV, Skelly T, Soloway MG, Tan D, Veluvolu U, Fan H, Hinoue T, Laird PW, Shen H, Zhou W, Bellair M, Chang K, Covington K, Creighton CJ, Dinh H, Doddapaneni HV, Donehower LA, Drummond J, Gibbs RA, Glenn R, Hale W, Han Y, Hu J, Korchina V, Lee S, Lewis L, Li W, Liu X, Morgan M, Morton D, Muzny D, Santibanez J, Sheth M, Shinbrot E, Wang L, Wang M, Wheeler DA, Xi L, Zhao F, Hess J, Appelbaum E. L. a. (2018). Oncogenic Signaling Pathways in The Cancer Genome Atlas. Cell.

[38] McDonald ER, de Weck A, Schlabach MR, Billy E, Mavrakis KJ, Hoffman GR, Belur D, Castelletti D, Frias E, Gampa K, Golji J, Kao I, Li L, Megel P, Perkins TA, Ramadan N, Ruddy DA, Silver SJ, Sovath S, Stump M, Weber O, Widmer R, Yu J, Yu K, Yue Y, Abramowski D, Ackley E, Barrett R, Berger J, Bernard JL, Billig R, Brachmann SM, Buxton F, Caothien R, Caushi JX, Chung FS, Cortés-Cros M, DeBeaumont RS, Delaunay C, Desplat A, Duong W, Dwoske DA, Eldridge RS, Farsidjani A, Feng F, Feng JJ, Flemming D, Forrester W, Galli GG, Gao Z, Gauter F, Gibaja V, Haas K, Hattenberger M, Hood T, Hurov KE, Jagani Z, Jenal M, Johnson JA, Jones MD, Kapoor A, Korn J, Liu J, Liu Q, Liu S, Liu Y, Loo AT, Macchi KJ, Martin T, McAllister G, Meyer A, Mollé S, Pagliarini RA, Phadke T, Repko B, Schouwey T, Shanahan F, Shen Q, Stamm C, Stephan C, Stucke VM, Tiedt R, Varadarajan M, Venkatesan K, Vitari AC, Wallroth M, Weiler J, Zhang J, Mickanin C, Myer VE, Porter JA, Lai A, Bitter H, Lees E, Keen N, Kauffmann A, Stegmeier F, Hofmann F, Schmelzle T, Sellers WR (2017). Project DRIVE: a compendium of cancer dependencies and synthetic lethal relationships uncovered by large-scale, deep RNAi screening. Cell.

[39] Barbieri I, Tzelepis K, Pandolfini L, Shi J, Millán-Zambrano G, Robson SC, Aspris D, Migliori V, Bannister AJ, Han N, De Braekeleer E, Ponstingl H, Hendrick A, Vakoc CR, Vassiliou GS, Kouzarides T (2017). Promoter-bound METTL3 maintains myeloid leukaemia by m6A-dependent translation control. Nature.

[40] Uddin MB, Roy KR, Hosain SB, Khiste SK, Hill RA, Jois SD, Zhao Y, Tackett AJ, Liu YY (2019). An N6-methyladenosine at the transited codon 273 of p53 pre-mRNA promotes the expression of R273H mutant protein and drug resistance of cancer cells. Biochem Pharmacol.

[41] Lin S, Choe J, Du P, Triboulet R, Gregory RI (2016). The m6A methyltransferase METTL3 promotes translation in human cancer cells. Mol Cell.

[42] Dominissini D, Moshitch-Moshkovitz S, Schwartz S, Salmon-Divon M, Ungar L, Osenberg S, Cesarkas K, Jacob-Hirsch J, Amariglio N, Kupiec M, Sorek R, Rechavi G (2012). Topology of the human and mouse m6A RNA methylomes revealed by m6A-seq. Nature.

[43] Kwok CT, Marshall AD, Rasko JEJ, Wong JJL (2017). Genetic alterations of m6A regulators predict poorer survival in acute myeloid leukemia. J Hematol Oncol.

[44] Graf R, Li X, Chu VT, Rajewsky K (2019). sgRNA sequence motifs blocking efficient CRISPR/Cas9-mediated gene editing. Cell Rep.

[45] Gisler S, Gonçalves JP, Akhtar W, de Jong J, Pindyurin AV, Wessels LFA, van Lohuizen M (2019). Multiplexed Cas9 targeting reveals genomic location effects and gRNA-based staggered breaks influencing mutation efficiency. Nat Commun.

[46] Verkuijl SA, Rots MG (2019). The influence of eukaryotic chromatin state on CRISPR–Cas9 editing efficiencies. Curr Opin Biotechnol.

[47] Listgarten J, Weinstein M, Kleinstiver BP, Sousa AA, Joung JK, Crawford J, Gao K, Hoang L, Elibol M, Doench JG, Fusi N (2018). Prediction of off-target activities for the end-to-end design of CRISPR guide RNAs. Nat Biomed Eng.

[48] Gutierrez B, Ng JW, Cui L, Becavin C, Bikard D. Genome-wide CRISPR-Cas9 screen in E. coli identifies design rules for efficient targeting. bioRxiv. 2018:308148. 10.1101/308148.

[49] Behan FM, Iorio F, Picco G, Gonçalves E, Beaver CM, Migliardi G, Santos R, Rao Y, Sassi F, Pinnelli M, Ansari R, Harper S, Jackson DA, McRae R, Pooley R, Wilkinson P, van der Meer D, Dow D, Buser-Doepner C, Bertotti A, Trusolino L, Stronach EA, Saez-Rodriguez J, Yusa K, Garnett MJ (2019). Prioritization of cancer therapeutic targets using CRISPR–Cas9 screens. Nature.

[50] Dwane L, Behan FM, Gonçalves E, Lightfoot H, Yang W, van der Meer D, Shepherd R, Pignatelli M, Iorio F, Garnett MJ (2021). Project Score database: a resource for investigating cancer cell dependencies and prioritizing therapeutic targets. Nucleic Acids Res.

[51] The Broad Institute. DepMap Portal Resources. 2021. https://depmap.org/portal/documentation/.

[52] R Core Team. R: A Language and Environment for Statistical Computing. Vienna; 2020. https://www.R-project.org/.

[53] Dowle M, Srinivasan A. data.table: Extension of ‘data.frame’. 2020. R package version 1.13.2. https://CRAN.R-project.org/package=data.table.

[54] Chang W. R6: Encapsulated Classes with Reference Semantics. 2020. R package version 2.5.0. https://CRAN.R-project.org/package=R6.

[55] Hart T, Brown KR, Sircoulomb F, Rottapel R, Moffat J (2014). Measuring error rates in genomic perturbation screens: gold standards for human functional genomics. Mol Syst Biol.

