# Supplementary material for: ACE: a probabilistic model for characterizing gene-level essentiality in CRISPR screens
Source: Genome Biol. 2021 Sep 23;22:278. doi: 10.1186/s13059-021-02491-z (PMC8459512; doi:10.1186/s13059-021-02491-z)
Supplement: Supplementary file 1 — Additional file 1 Supplemental figures and analysis. [file 13059_2021_2491_MOESM1_ESM.pdf]

Supplemental Figures and Discussion for ‘ACE: A Probabilistic Model for Characterizing Gene-Level Essentiality in CRISPR Screens’

Elizabeth Hutton, Chris Vakoc, Adam Siepel

September 8, 2021

Contents

1 Background **2**

2 Model Overview **3**

3 Simulation Study **4**

4 Benchmarking of Absolute Essentiality Predictions **5**

5 Identification of Essential Genes **6**

6 Identification of Differentially Expressed Genes **7**

7 Test for Non-Oncogene Addictions in TP53 Mutants **15**

8 Run Time Analysis **19**

1 Background

Method	Directly Models Count Data	Estimates Essentiality	Tests for Differences Between Subtypes
ACE	✓	✓	✓
Average Fold Change (AFC)	✗	✓	✗
BAGEL (Hart et al. 2015)	✗	✗	✓
JACKS (Allen et al. 2019)	✗	✓	✗
MAGeCK-RRA (Li et al. 2014)	✗	✗	✗
CERES (Meyers et al. 2017)	✗	✓	✓
ScreenBEAM (Yu et al. 2015)	✗	✓	✗
CB2 (Jeong et al. 2019)	✓	✗	✗
PinAPL-Py (Spahn et al. 2017)	✓	✗	✗
MAGeCK-MLE (Li et al. 2015)	✓ *	✓	✗

*depleted read counts only

Figure S 1: Methods for Identifying Differential Essentiality in CRISPR Screens. Many methods have been developed to analyze CRISPR screening data, but none are designed to test for differential essentiality between sample subtypes as a part of their inference framework. Some, such as BAGEL and CERES, test for significant differences in essentiality after parameter inference, but do not test whether the data is best described by a single essentiality parameter ('Tests for Differences Between Subtypes' column). Directly modeling count data, as opposed to relying upon summary statistics such as log-fold change, enables ACE to adapt to variations in experimental design, and leverage information from all available data ('Directly Models Count Data' column). Providing a quantification of essentiality, as opposed to fitting a binary essential/nonessential classification, enables researchers to more intuitively estimate phenotype ('Estimates Essentiality' column).

2 Model Overview

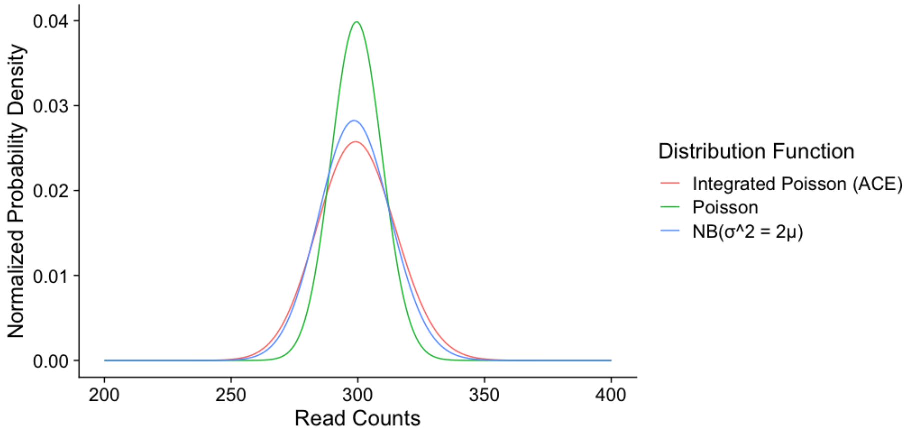

Figure S 2: Illustration of Dispersion of ACE Distribution Function. Read count data is known to have a high dispersion similar to a Negative Binomial distribution [**?**]. The values shown here reflect the probability density of different values of read counts for a single guide in a CRISPR screen with a mean of 300, as determined by the product of sampling from the infection, initial sequencing, and final sequencing stages with no effect parameters. Shown are the probability densities described by modelling these sampling processes with a Poisson distribution with point estimates of the mean (green), a Negative Binomial distribution with point estimates of the mean and a variance equal to twice the mean (blue), and a Poisson distribution integrated over all possible infected cells, as per the ACE model, resulting in a probability density (red) similar to the Negative Binomial.

3 Simulation Study

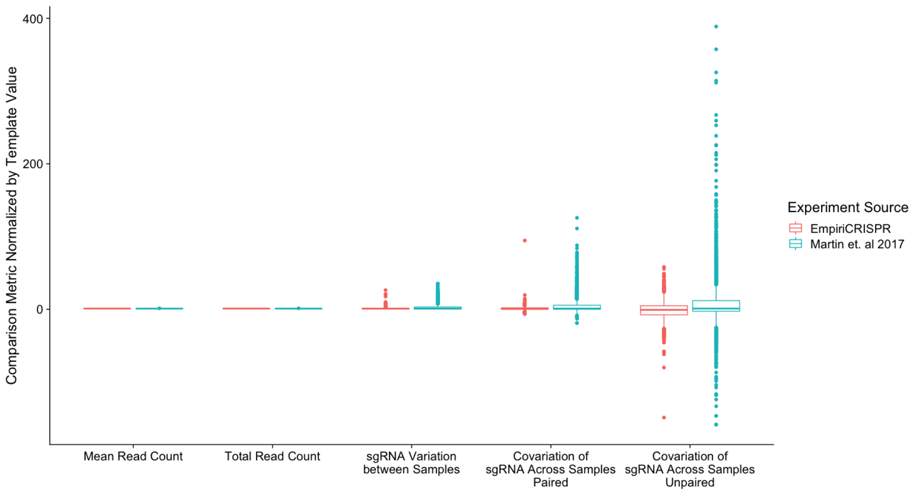

Figure S 3: Simulated Data for Evaluation of ACE Performance. The genome-wide CRISPR screen performed by Martin et. al 2017 [?] was used as our EmpiriCRISPR template, as this work sequenced the sgRNA master library used as well as the initial and final samples. The sequenced master library provided the initial sgRNA abundances in the simulation, and all metrics shown were calculated in the initial and depleted counts. Shown are approximately 2300 guides targeting globally nonessential genes, as identified using ENCODE expression data (see Methods), and a randomly selected matching subset of nonessential sgRNA from simulated read counts. Mean and total read counts were calculated within each sample, while variation and covariation metrics were calculated for each guide across samples. 'Paired' indicates covariance was calculated between initial and depleted samples, with each sgRNA paired with the depleted sample produced by the same master library transfection; 'Unpaired' have been paired with samples from a different transfection replicate.

4 Benchmarking of Absolute Essentiality Predictions

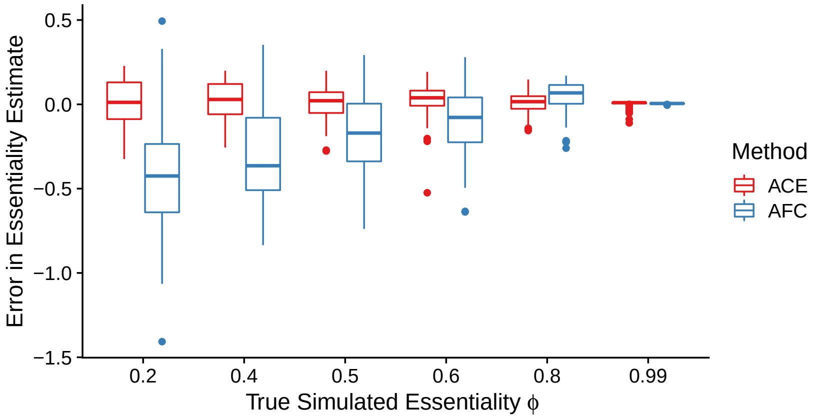

Figure S 4: Error in Estimation of Gene Essentiality. Gene essentiality ϕ_G was calculated using ACE and average fold change (AFC; see Methods) across three replicates of simulated count data. 300 genes were simulated at each essentiality level shown; results from an additional 3,150 nonessential genes are not shown.

5 Identification of Essential Genes

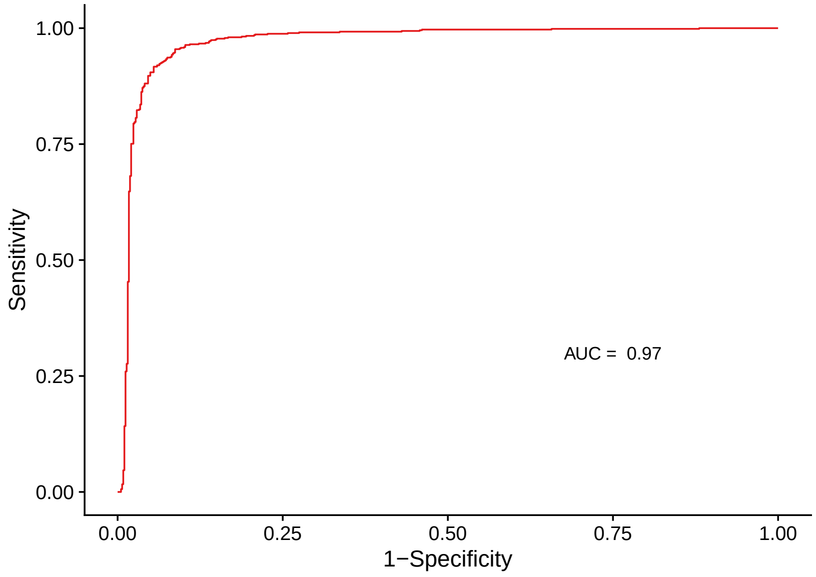

Figure S 5: ACE Classification of Established Essential and Nonessential Genes in Achilles DepMap CRISPR Screens. All 220 CRISPR screens performed in 92 different NSCLC Adenocarcinoma cell lines in the Achilles DepMap project [?] were used by ACE to estimate the likelihood of essentiality of 688 essential genes.

6 Identification of Differentially Expressed Genes

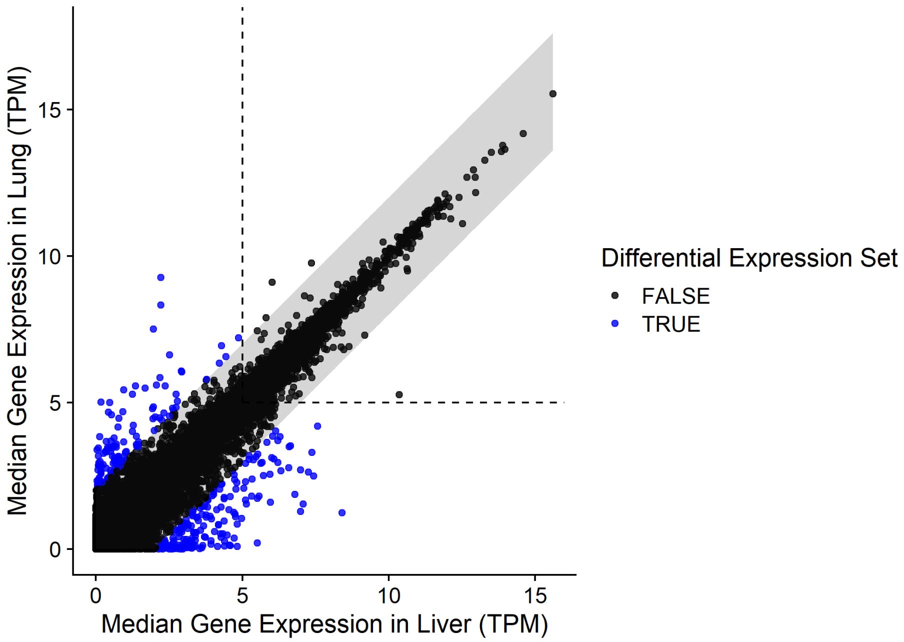

Figure S 6: Differentially Expressed Genes for Differential Essentiality Test Set. Genes selected for differential expression test set according to median CCLE gene expression data in lung and liver samples. Shaded region indicates threshold for gene expression differences. Dotted lines show cutoff for genes highly expressed in both tissue panels. 492 genes shown in blue were selected to test for differential essentiality based on their tissue-biased expression.

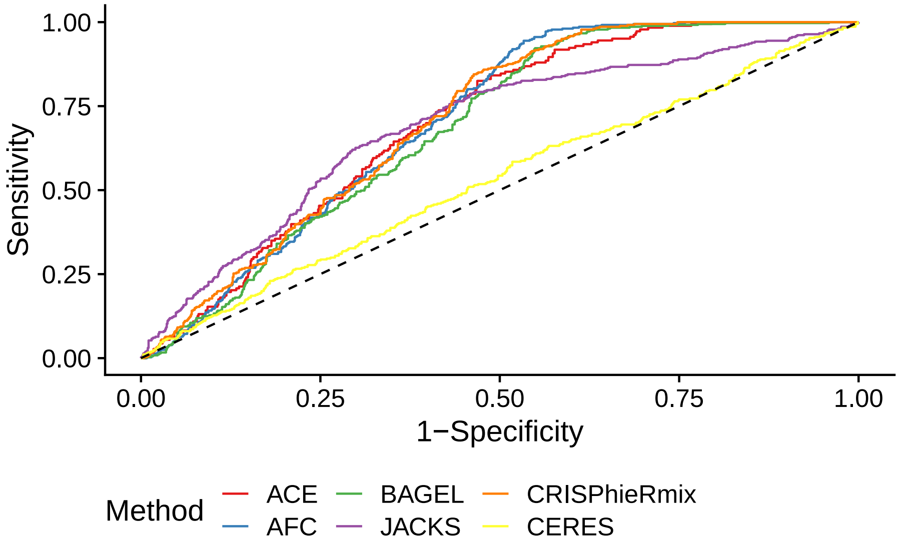

Figure S 7: Identification of Differentially Expressed Genes in Achilles DepMap CRISPR Screens. Gene classification performance based on estimates for differential essentiality. The 'CERES' test statistic was derived from a t-test using precomputed CERES scores for the relevant DepMap samples, as used in [**?**]; other methods were used as previously described.

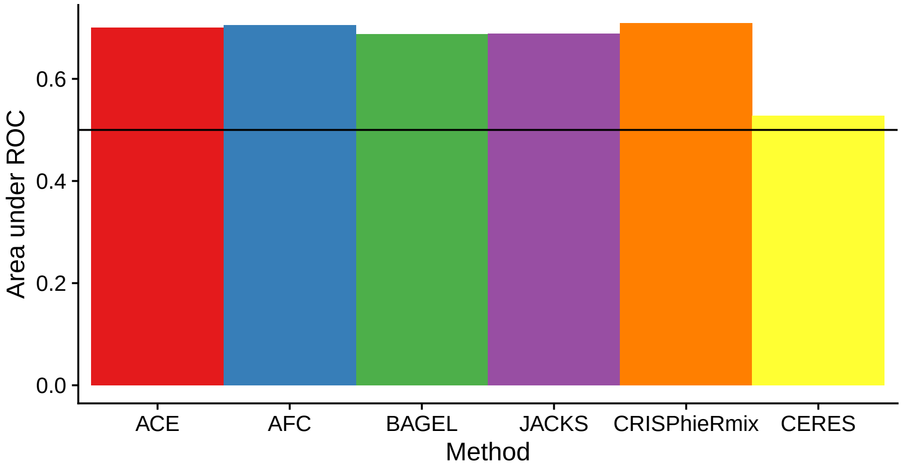

Figure S 8: AUC of Identification of Differentially Expressed Genes in Achilles DepMap CRISPR Screens. 42 CRISPR screens from liver and lung tissue panels were selected. 1713 genes were selected using CCLE gene expression data into constitutively expressed (N=698), silent (N=594), and tissue-specific (N=492) categories, and tissue-specific genes were identified (AUC: CRISPhieRmix = 0.709, AFC = 0.705, ACE = 0.701, JACKS = 0.688, BAGEL = 0.687, CERES = 0.528). See Methods for discussion of test statistics for each method. Horizontal line shown for AUC = 0.5.

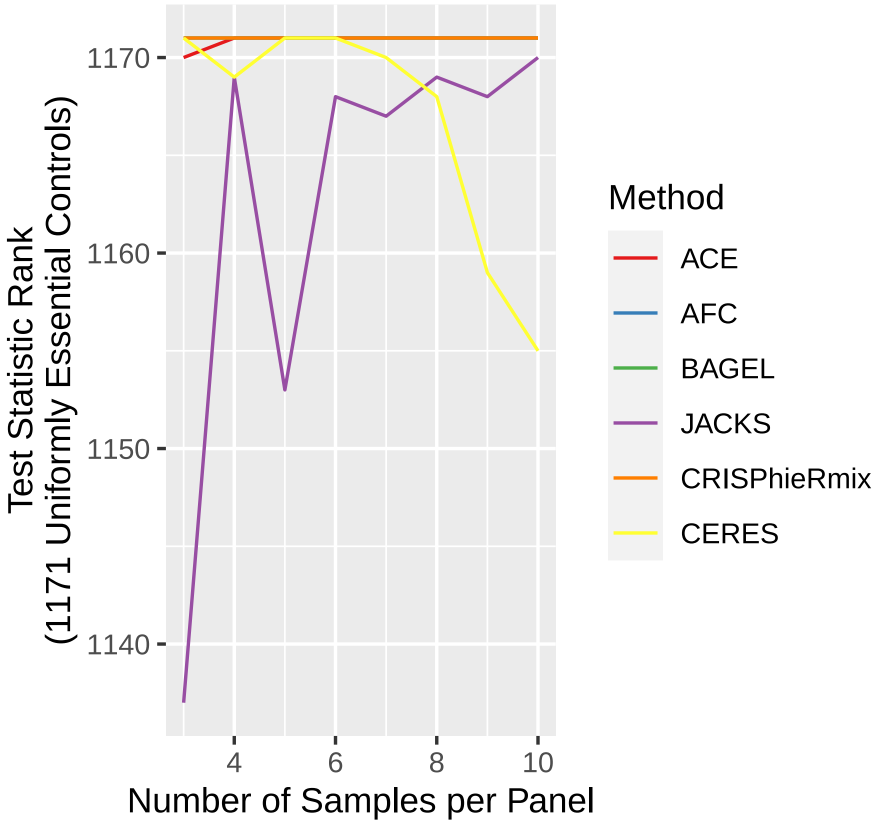

Figure S 9: Identification of Known Genotype-Specific Essentiality. Shown is the rank of *MDM2*, a negative regulator of *TP53*, by its differential essentiality test statistic between wildtype and damaged *TP53* sample panels. Ranking is relative to 1,171 uniformly essential or nonessential genes from our previously described negative and positive control sets; a subset of 50 from each of these sets were provided to methods as annotated controls.

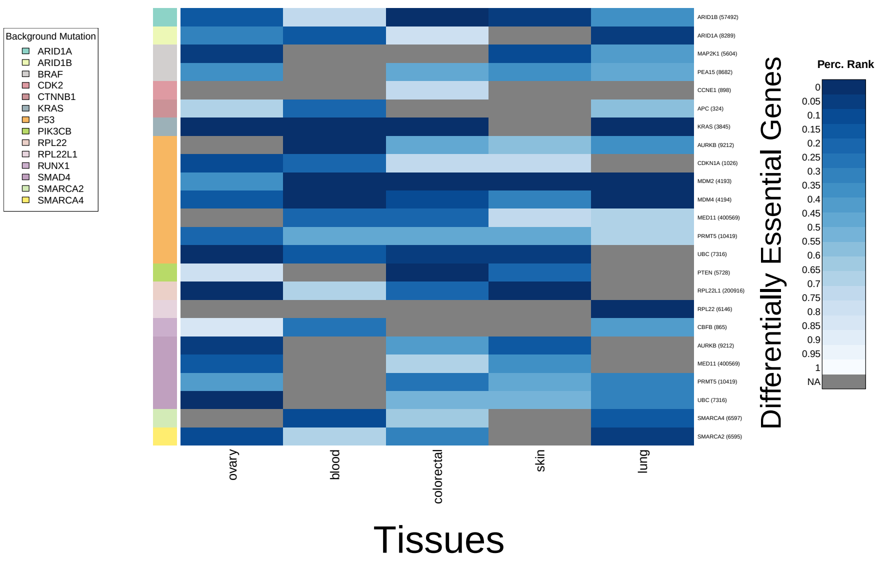

Figure S 10: ACE Identification of Essential Genes in Low-sample Analyses. ACE ranking of known differentially essential genes relative to 1000 uniformly essential control genes (500 essential, 500 nonessential from our previously described negative and positive control sets). A one-sided test was performed for each gene based on the reported gain or loss of essentiality (see Methods). Lower percentile rank indicates a stronger differentially essential test statistic. Results are shown for differentially essential genes (rows) in five tissue types (columns), with three samples in each of the test and control genetic background panels. These panels consisted of cell lines with and without mutations in genes shown on the left. Gray conditions reflect instances with inadequate numbers of samples carrying the required background mutation, or cases where there were insufficient read counts to determine the expected direction of essentiality. The median rank of a differentially essential gene was 0.243; results are shown even if they were not significant.

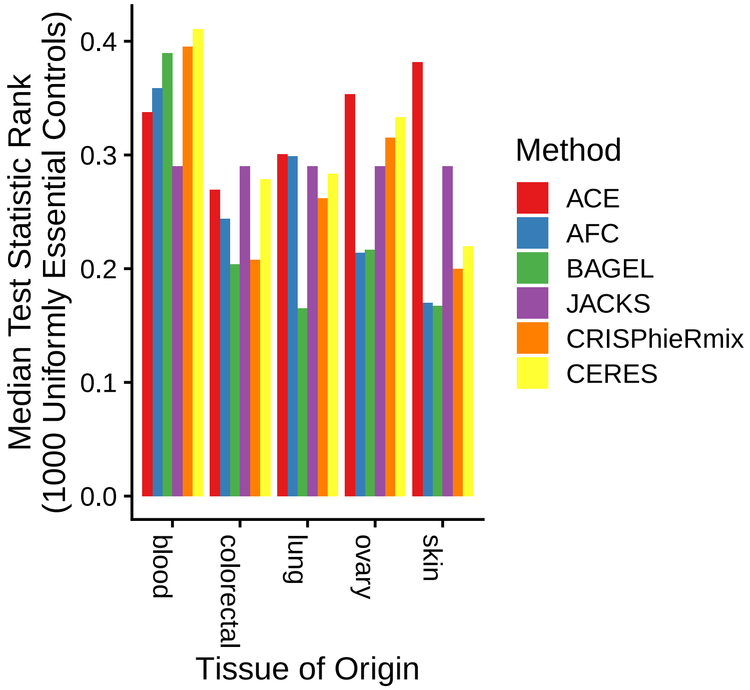

Figure S 11: Comparison of method rankings of differential essential genes by tissue. Results are shown for all genes and genetic backgrounds described in Figure S10; no directional testing was used to calculate ranks.

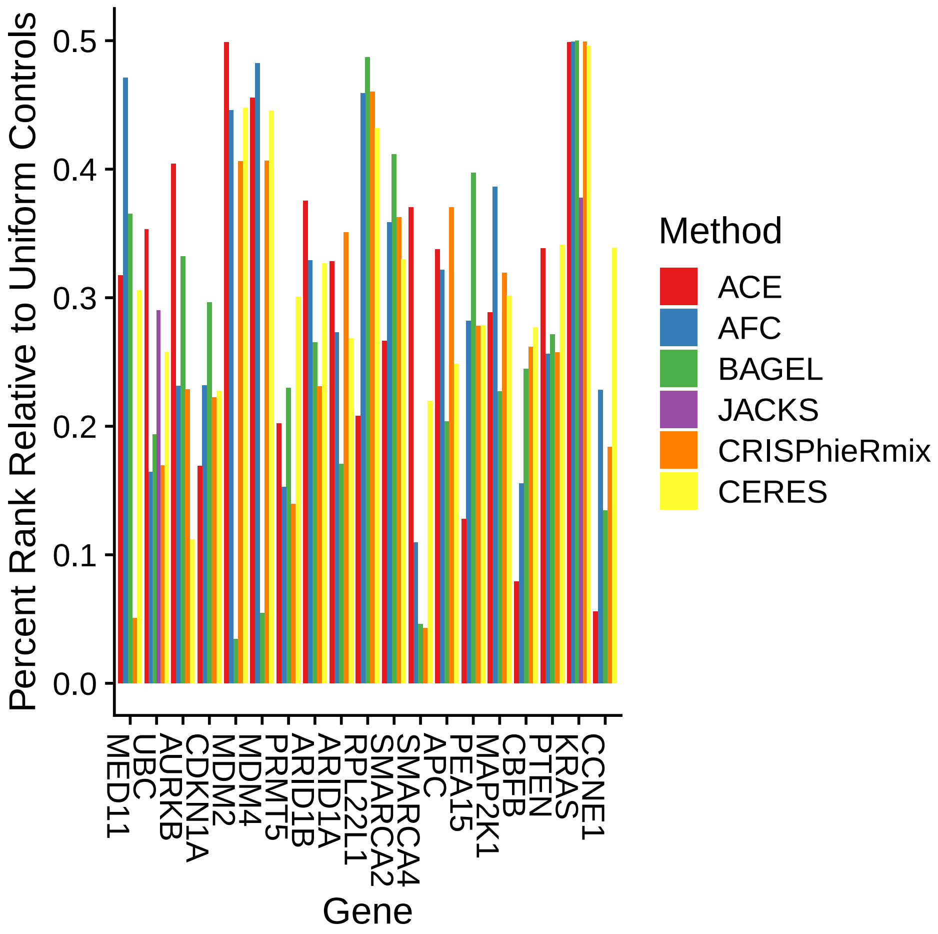

Figure S 12: Comparison of method rankings of differential essential genes by gene. Results are shown for all tissues and genetic backgrounds described in Figure S10; no directional testing was used to calculate ranks.

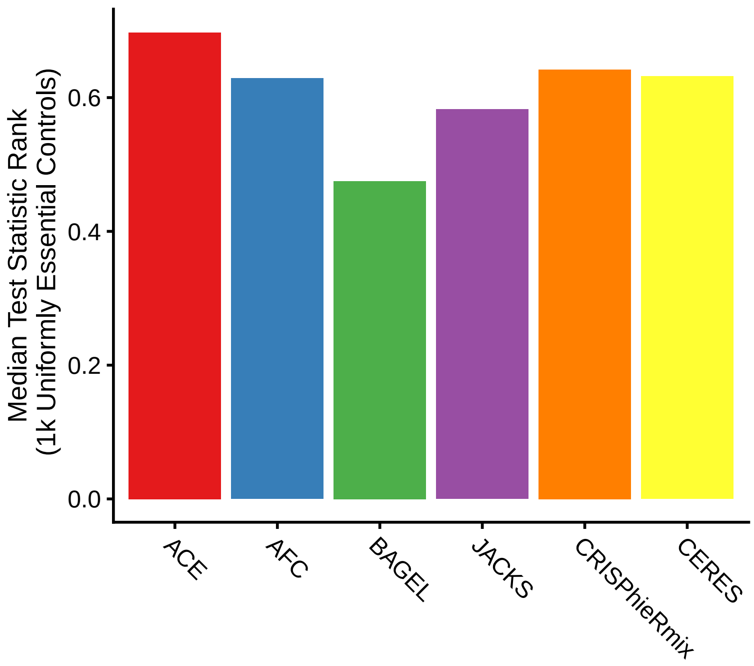

Figure S 13: Comparative Identification of Essential Genes in Low-sample Analyses. Median ranking using one-sided test of 19 differentially expressed genes and their 14 associated genetic backgrounds across five tissue types from DepMap's Project Achilles. Ranking is relative to 500 uniformly essential and 500 uniformly nonessential genes from our previously described negative and positive control sets.

7 Test for Non-Oncogene Addictions in TP53 Mutants

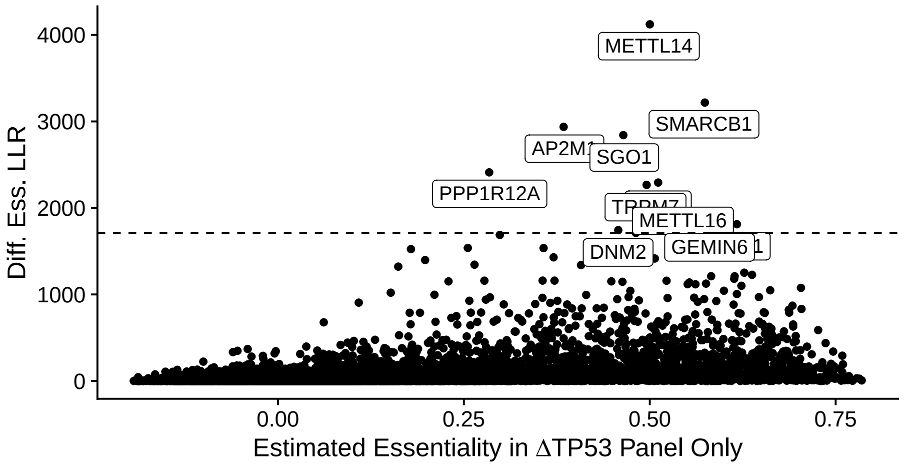

Figure S 14: Genes Essential in NSCLC Adenocarcinoma cell lines with a 'damaging' mutation in *TP53*. All genes evaluated by genome-wide CRISPR screens in NSCLC cell lines by the DepMap Project Achilles are shown. On the x-axis is the ACE estimate of essentiality in cell lines with a nonsense or frameshift mutation indicated as 'damaging' in *TP53* (ΔTP53 Panel; see Methods). On the y-axis is the test statistic indicating whether this essentiality is significantly different from cell lines with wildtype *TP53*, calculated with a log likelihood ratio test comparing a shared versus a unique essentiality between the two sample panels. The horizontal line indicates the Bonferroni-correct empirical p-value <0.05.

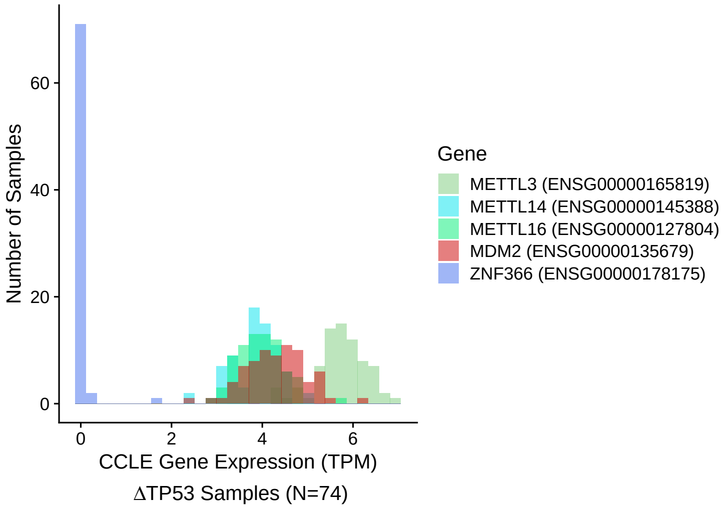

a

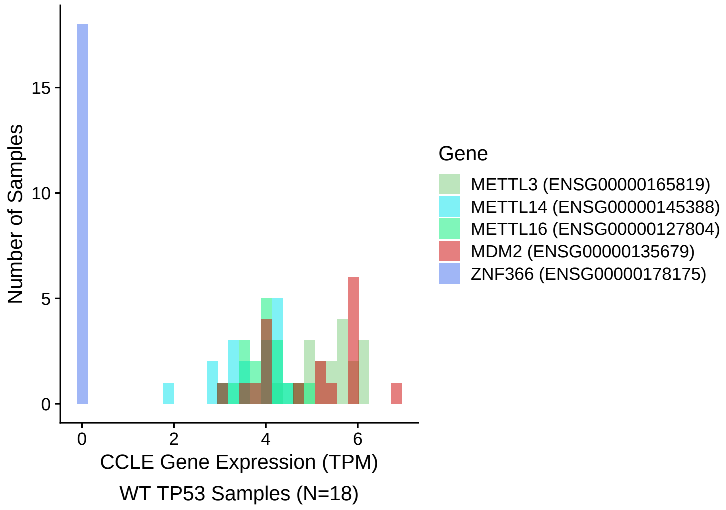

b

Figure S 15: Expression Levels of Differential Essentiality Candidates. CCLE reported expression levels of differential essentiality candidates *METTL3*, *METTL14*, and *METTL16* in cell lines used by the Achilles DepMap project with annotated nonfunctional **(a)** and presumed functional **(b)** variants of *TP53* [**?**, **?**, **?**]. *MDM2* is shown as a positive control for gene essentiality; *ZNF366* as a negative control.

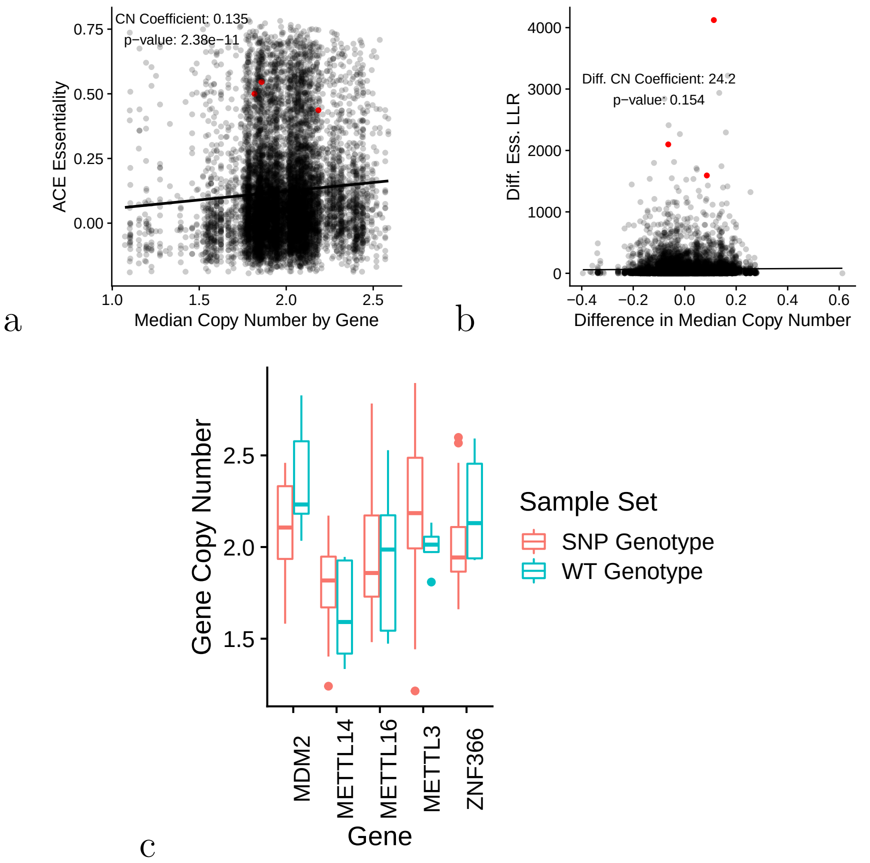

Figure S 16: ACE Essentiality Estimates Avoid Copy Number Sensitivity by Pooling Across Sample Panels. **(a)** The ACE estimated essentiality of each gene (y-axis), with the median copy number of that gene across all NSCLC Adenocarcinoma samples with a mutation in *TP53* used to infer the essentiality value (x-axis). **(b)** The differential essentiality test statistic of each gene between wildtype and Δ*TP53* cell lines, determined by ACE. On the x-axis is the difference in median copy number between the compared panels of cell lines. In red are *METTL3, METTL14* and *METTL16.* The black line indicates the best linear relationship given a gaussian error model. **(c)** Copy number variation across each sample panel for the three candidate differentially essential methyltransferases, with *MDM2* as a positive control, and *ZNF366* as a negative control for differential essentiality. The range in copy number largely overlaps for all genes in these sample panels.

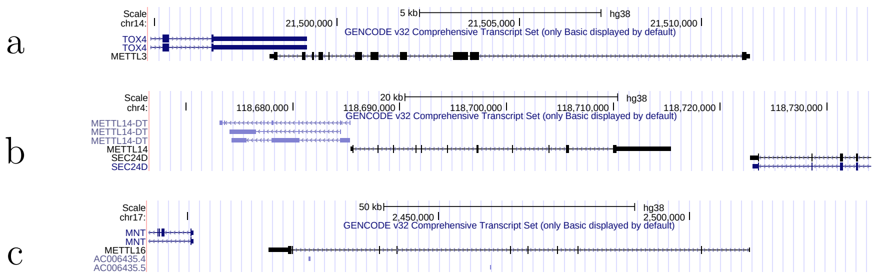

Figure S 17: Differentially Essential Gene Candidates Independent of Flanking Genes. The majority of METTL3 **(a)**, and all of METTL14 **(b)**, and METTL16 **(c)** do not overlap with other coding genes. Image from the UCSC genome browser.

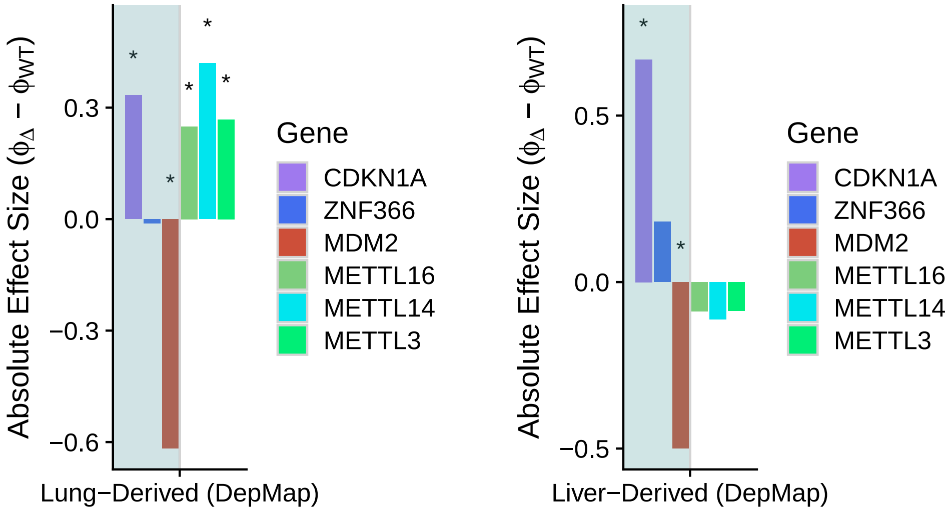

a

b

Figure S 18: Gene Candidates Not Differentially Essential in Liver Samples. Absolute effect size of essentiality in samples with and without mutations in TP53. Performed in 200 NSCLC lung samples, and 42 liver samples from the Broad's Project Achilles with the DepMap Consortium. Asterisk indicates empirical p-value < 0.05 for differential essentiality. *CDKN1A* and *MDM2* are shown as examples of positive controls for differential essentiality, and *ZNF366* as an example of a nonessential control (the full set of negative control genes used in the ACE analysis is described in Methods). The absence of a strong *TP53*-dependent effect in liver-derived samples may reflect the high frequency of gain-of-function mutations in the *Wnt* pathway, namely in *CTNNB1*, which are tenfold more likely with a wildtype *TP53* background in liver-derived samples than in lung [**?**].

8 Run Time Analysis

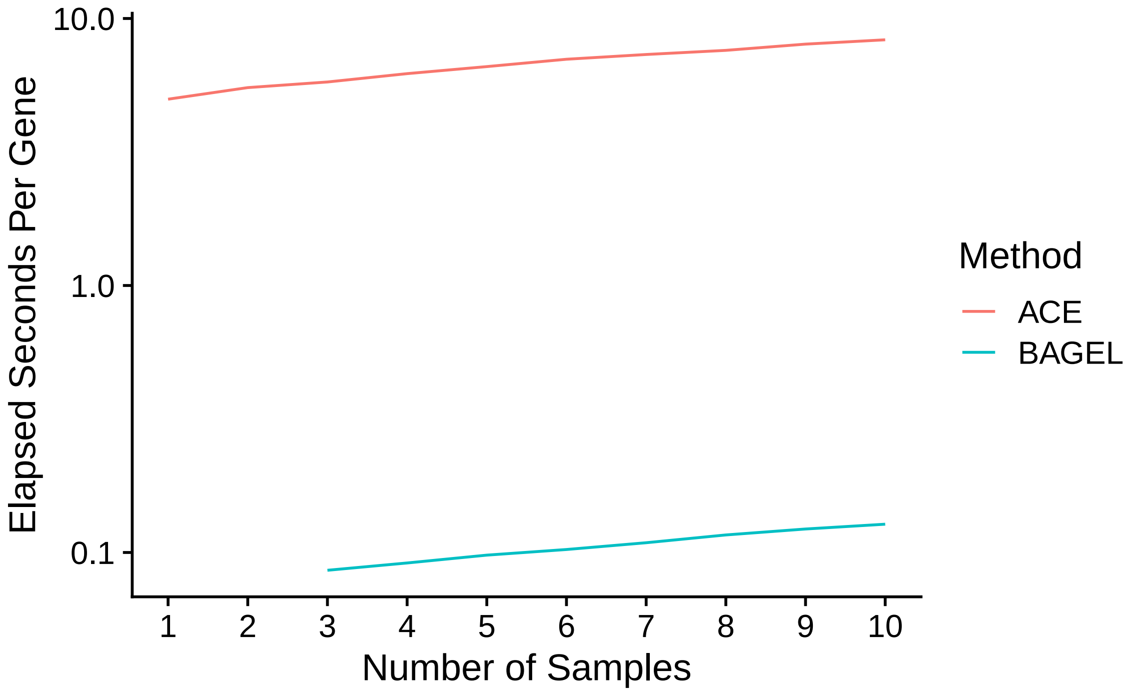

Figure S 19: Run Time Analysis For a Single Gene in ACE and BAGEL. Results are shown for the elapsed time to evaluate the essentiality of one gene with four sgRNA from the DepMap Consortium Achilles Project, as measured by the R 'benchmark' package on a Windows operating system with an AMD Ryzen 5 2600 Six-Core Processor (only one core is used for the estimation of a single gene's parameters). ACE results are shown as the median time from the separate analysis of five random genes; BAGEL results are the average elapsed time per gene from the joint analysis of 1000 genes. ACE can analyze all genes in parallel, while all genes must be analyzed by BAGEL on a single thread.
