# Supplementary material for: ACE: a probabilistic model for characterizing gene-level essentiality in CRISPR screens
Source: Genome Biol. 2021 Sep 23;22:278. doi: 10.1186/s13059-021-02491-z (PMC8459512; doi:10.1186/s13059-021-02491-z)
Supplement: Supplementary file 3 — Additional file 3 Review history. [file 13059_2021_2491_MOESM3_ESM.docx]

Review History

**First round of review**

**Reviewer 1**

**Were you able to assess all statistics in the manuscript, including the appropriateness of statistical tests used?**

Yes, and I have assessed the statistics in my report.

**Were you able to directly test the methods?**

No

**Comments to author:**

Hutton et al. proposes a novel hierarchical model to estimate the essentiality of genes across CRISPR-Cas9 single-gene perturbation screens and identify differential gene depletions across two groups of samples. The paper considers a set of latent variables representing the true number of infected cells at early/late time points, which are then treated as priors for the observed read counts. The deterministic relationship between the latent variables captures the gene essentiality/efficacy. The authors use MLE (maximum likelihood estimation) as the inference procedure which involves integration over latent variables and a coordinate ascent optimization. The MLE is different from classical point estimates of likelihood due to the hierarchical structure that reduces bias in variance/overfitting. Overall, the model is novel, the inference procedure while not new is straightforward, and the differential analysis is robust. It is important to note that the proposed method only reflects an 'average' essentiality across all samples, not sample-specific essentiality. The method comparison, which is limited to simulated data, suggests that identification of differential essential genes between two sets of samples outperforms other methods, however, other methods slightly better identify essential genes in one set of samples. This improvement resulted in a number of interesting candidates for non-oncogene addiction in the absence of functional TP53. While this is an interesting approach to the analysis of CRISPR viability screens several issues should be addressed.

Major concerns

1. It would be important to compare the computational time required for the author's proposed method to other methods since 1) the integration over latent variables is expensive as it does not utilize the marginal posterior and 2) the use of R's optim per variable might be time consuming in high-dimensional settings. This of course would be important as data set sizes increase (including currently available datasets).

2. There should be a stronger justification/analysis for the choice of Poisson distribution of the read counts. Specifically, the authors assume a Poisson distribution for the observed read count of each sgRNA (x and y) as well as a Poisson prior to account for the variability of the mean. Given that read counts are not sparse and we often see a reasonable number of counts per sgRNA, a Negative Binomial might also be a reasonable choice, or another exponential family distribution that would allow the mean to be different from variance and thus not lead to an underestimation of the true variance. The paper shows in the Supplementary Figure S2, that the Integrated Poisson resembles the underlying count distribution across all sgRNAs in a sample. However, I am concerned about this assessment since 1) count distribution across all sgRNAs can be different from the count distribution for any specific guide across replicates, which is modeled by the Poisson and 2) Supplementary Figure S2 is just specific to one type of library design, whereas the CRISPR-Cas9 systems can vary from one lab to another.

3. The authors use gamma parameters to connect the latent variables to observed counts. The gamma parameters are quite important, and the authors do not estimate them through MLE, but rather through a pre-processing step. The pre-processing estimations are reasonable; however, without any evidence/analysis, the paper claims this estimation is adequate compared to treating them as variables in MLE. Supportive evidence and/or analysis should be provided to justify the use of pre-processing estimates.

4. The differential analysis is based on a likelihood ratio test of (Test times Control)/(combined Test and Control). This is a reasonable and adequate statistical test. It would be of interest to the readers to see this generalized to more than two group comparisons, e.g. borrowing ideas from intersection-union test (Casella, G. & Berger, R. L. Statistical Inference, 2002). This might be helpful for characterization of tumor types with high degree of heterogeneity in mutation profiles or other genomic features.

5. The comparison of this new method using the simulated data shows that JACKS, which utilizes a variational Bayes framework, outperforms the proposed ACE in the detection of essential genes. The differential assessment shows ACE outperforms existing methods and I consider this as a novel space as proposed by Hutton et al. However, there are two major concerns. First, there are other competitive algorithms such CRISPhieRmix and CERES that are not included in the Figs. 2 and 3. Hence, such additional methodologies should be included in the comparative evaluation. Second, while I appreciate the simulation results, method comparison should also cover real datasets such as Achilles (i.e. Broad DepMap) or Sanger CRISPR-Cas9 dependency datasets. To build controls, one can treat genes that are non-expressed across the majority of CCLE cell lines as negative controls, and known/published essential genes found from Achilles or DRIVE RNAi datasets as positive controls. To show the robustness of differential analysis, one could further categorize the positive controls into strong, modest, or weak groups according to the distribution of gene essentiality (for instance CERES scores) across all cell lines.

6. In "Identification of Essential Genes" subsection, the authors employ pan-essential and non-expressed genes as positive and negative controls. These genes should be essential or non-essential regardless of cell line profiles. Hence, the ACE should not be limited to only NSCLC cell lines, rather expanding to all Achilles cell lines to evaluate ACE performance across a wider range of samples with more variability in essentiality levels. Moreover, there is no need to limit the analysis to only positive and negative control genes whereas the other genes can serve as another category (i.e. cell-line/subtype specific dependencies) to evaluate performance. In this category, the essentiality level should be lower than positive controls and potentially higher than negative controls.

7. In "Test for Non-Oncogene Addictions in TP53 Mutants" subsection (Fig. 5d), If all Achilles cell lines are used, a simple correlation between the dependency of TP53 and every other gene shows MDM2 is among top 5 well-correlated genes. Thus, there needs to be a method comparison to evaluate the sensitivity of differential genes with respect to the number of samples. This could elucidate in what cases ACE is strongly outperforms heuristic approaches.

8. The paper proposes the mRNA methyltransferases as new candidates based on a limited dataset, and it makes sense, and would seem easy to confirm/validate this finding in separate datasets. For instance, the Sanger data and/or another tumor type in Achilles datasets would be independent datasets that should clearly reproduce the results.

Other comments

1)	The paper uses MLE with a hierarchical model, however, the word "Bayesian" are often used, e.g. "hierarchical Bayesian model" throughout the paper. There is no Posterior estimation involved, and thus "Bayesian" should not be used.

2)	In "Benchmarking of Differential Essentiality Predictions" subsection, why not using limma on the gene-level values (i.e. w) inferred by JACKS to obtain differential genes? I am not sure the ratio of p-values for the test and control is an appropriate statistical test.

3)	Fig. 5d is confusing. I assume the asterisk next to MDM2 rank value shows the ranking when the test is wildtype TP53 while for the other rows the test is delta TP53? This needs a better representation since reader might think MDM2 is dependent in delta TP53. Also, it is worth showing where the CDKN1A's rank stands in wildtype TP53.

4)	The explanation of empiriCRISPR in Method section, the package to generate simulation data, is not clear.

5)	I could not find the ACE/empiriCRISPR packages at https://github.com/CshlSiepelLab. The package should be available, along with a manual/vignette.

**Reviewer 2**

**Were you able to assess all statistics in the manuscript, including the appropriateness of statistical tests used?**

No, I do not feel adequately qualified to assess the statistics.

**Were you able to directly test the methods?**

No

**Comments to author:**

In the study by Hutton et al, the authors describe a probabilistic framework ACE for the analysis of CRISPR screens. They show using simulated and publicly available CRISPR-Cas9 screening data that ACE is suitable for calling gene essentiality and can identify differential essentiality. Using ACE, the authors report a differential essentiality for RNA methyltransferases in the setting of TP53 mutant lung cancer cell lines.

The ACE analytical framework is an elegant computational approach and the incorporation of additional sources of experimental variation into analytical models could prove useful for modelling gene essentialities from CRISPR Cas9 screens. Nonetheless, to my mind, the authors do not make a compelling argument for the added value of the ACE framework above and beyond available models such as BAGEL and JACKS, or the use of AFC. Any additional gains of ACE beyond these other analytical frameworks seems modest and incremental. The inclusion of a likelihood ratio test is an additional feature of ACE, but again it is unclear of the added value compared to approaches such as CERES or simple statistical test to assess differential essentiality. The manuscript would benefit from a clearer description of the benefits that distinguish the ACE framework and the specific application where it has particularly utility beyond other approaches.

Specific comments:

1. What are the applications or situations where the ACE framework would be most beneficial, above and beyond existing approaches? Would it for instance, be particularly useful for modelling low quality data where the fitness effect might be relatively modest, or sample variation is high? Would it be particularly useful for integrating data generated from different screens using different methodologies by accounting for additional experimental factors? Practical examples using 'real' data should be provided.

2. Copy number associated bias in gene fitness effects is a major confounder for Cas9 screens. Several methods have been developed to correct this effect (PMID: 29083409 and PMID: 30103702). A failure of ACE to systematically account for this effect is concerning and a limitation, and there is clearly an effect in their data (Figure S8a). Lung cancer is primarily driven my SNPs. Have the authors evaluated ACE in tissues types where copy number alterations are much more prevalent, such as breast or ovarian cancer cell lines, and much more likely to be have a pronounced confounding effect on the results?

3. Why have the authors excluded the number of base mismatches as a feature when determining sgRNA efficiency (and only included GC content)? This would seem important, especially when the mismatch is proximal to the PAM sequence.

4. Related to Figure 4. How does the performance of ACE compare to JACKS or BAGEL for the identification of essential genes when using the DepMap data? Also, for Figure 4a, can the authors comment on why so many genes appear to be classified as having decreased estimated essentiality?

5. The authors demonstrate that ACE can detect differential essentiality in test and control samples, using TP53 and KRAS in NCSLC as examples. The number of lung cell lines screened is large (n = 92) and these mutations are very prevalent in this tissue. How does ACE perform when working with smaller sample sizes which are typically the case for other tissue lineages and mutational events?

6. The authors argue the identification of RNA methyltransferases as a differential essentiality in TP53 mutant cells demonstrates the advantages of the ACE framework. While this observation is interesting, the finding is not validated with further replication studies to confirm the differential dependency, and no mechanistic insight is provided. Further, it is unclear why this difference is observed, except that they are using a different analytical framework. Thus, while the observation is interesting, and there are circumstantial evidence supporting the observation, it does not provide evidence that one approach is superior or more informative than the other without further insight.

7. Large studies reporting CRISPR screens in cell line panels from the Sabatini lab (28162770) and Sanger Institute (PMID: 30971826) should be cited in the introduction.

**Reviewer 2**

**Were you able to assess all statistics in the manuscript, including the appropriateness of statistical tests used?**

Yes, and I have assessed the statistics in my report.

**Were you able to directly test the methods?**

No

**Comments to author:**

This study by E. Hutton et al. introduces ACE - a novel statistical framework and software package for analysis of CRISPR screens. ACE applies probabilistic modeling and inference for identification of essential genes and maximum-likelihood estimation of gene essentiality from CRISPR negative selection screens. A main novelty of ACE is its inherent support for identification of differential essentiality, that is - genes that are essential only in a specific biological context. Additionally, the study developed a new method for simulation of CRISPR screen data - empiriCRISPR - generating, with minimal probabilistic assumptions, count data that closely resemble real one. Using simulation, ACE compares well with state-of-the-art tools.

The likelihood ratio tests implemented in ACE support the detection of both absolute and differential essentiality, the latter is ran on groups of samples designated as "control" and "test" ones. ACE's test for differential essentiality poses a marked advance in the field as it allows systematic identification of context-dependent essential genes from CRISP screens. This utility of ACE is demonstrated by a very intriguing finding detected on publicly available CRISPR-screen data in lung adenocarcinoma, indicating enhanced essentiality of RNA m6A methyltransferases in p53-mutant cancer cells.

Overall, it is a very nice and well-written study that presents a novel tool with several marked merits that make a substantial contribution to the analysis of CRISPR screens.

I only have a few comments:

1. As biological validation is out-of-scope of this computational/methodological study, the authors should strive to further substantiate their findings by additional analyses. The analysis presented in the manuscript was carried out on lung adenocarcinoma cell lines. (a) Does MDM2 turn essential only in p53-wt background in cell lines of other cancer types? (b) Do METTL14/16/3 show enhanced essentiality in p53-Mut background in cell lines of other cancer types? The indication of these METTLs as candidates for non-oncogene addiction in p53-Mutant cancer is very interesting. However, the support given for this observation is not strong (their expression levels (Fig 5c and FigS7)). Finding this effect in additional cancer types can significantly strengthen this result.

2. The usefulness of ACE package is largely dependent on the availability of a detailed user-friendly manual. I couldn't find ACE (nor empiriCRISPR) at the given link (https://github.com/CshlSiepelLab).

3. Fig. 4a. The scatter of the negative controls shows a branch to the right-top above significance threshold. Are these genes characterized by any specific biological annotation? Are they in fact true positives?

4. Fig S8. The title argues that ACE essentiality estimates avoid copy number bias. However panel (a) shows a significant overall correlation between copy number and ACE essentiality.

**Authors Response**

**Point-by-point responses to the reviewers’ comments:**

Reviewer #1

Major Comments:

1.	It would be important to compare the computational time required for the author's proposed method to other methods since 1) the integration over latent variables is expensive as it does not utilize the marginal posterior and 2) the use of R's optim per variable might be time consuming in high-dimensional settings. This of course would be important as data set sizes increase (including currently available datasets).

*We have included a figure illustrating the time required to execute ACE on data for a single gene (Figure S12). We find that ACE is indeed significantly slower than other methods: the evaluation of each gene takes a few seconds, while the next slowest method, BAGEL, requires tenths of a second per gene. Importantly, however, ACE can be executed over all genes in parallel on a computing cluster. Overall, a typical ACE analysis of a genome-wide CRISPR screen takes about 30 minutes with ~50 threads, which should not be prohibitive for most applications. Notably, we find that the execution time increases linearly, or even slightly less than linearly, with the data set size, suggesting good scalability to large datasets, particularly when a computer cluster of significant size is available.*

2.	There should be a stronger justification/analysis for the choice of Poisson distribution of the read counts. Specifically, the authors assume a Poisson distribution for the observed read count of each sgRNA (x and y) as well as a Poisson prior to account for the variability of the mean. Given that read counts are not sparse and we often see a reasonable number of counts per sgRNA, a Negative Binomial might also be a reasonable choice, or another exponential family distribution that would allow the mean to be different from variance and thus not lead to an underestimation of the true variance. The paper shows in the Supplementary Figure S2, that the Integrated Poisson resembles the underlying count distribution across all sgRNAs in a sample. However, I am concerned about this assessment since 1) count distribution across all sgRNAs can be different from the count distribution for any specific guide across replicates, which is modeled by the Poisson and 2) Supplementary Figure S2 is just specific to one type of library design, whereas the CRISPR-Cas9 systems can vary from one lab to another.

*We appreciate the concern about overdispersed read counts, which we perhaps addressed too cavalierly in our first submission. In fact, we have considered this issue in some detail, and we have attempted to provide a clearer justification for our approach in the revised manuscript.*

*As it happens, our initial design for ACE did indeed use a Negative Binomial distribution for read counts, allowing the gene-wise dispersion parameter to vary as a function of the mean, as in DEseq2 (Love et al., 2014). However, we found this version of the model awkward and computationally expensive to fit to the data, and we began to experiment with the simpler Poisson model for comparison. Somewhat to our surprise, we found that the Poisson version performed as well or better than the Negative Binomial version, considering the challenges of fitting the model. The read counts in real data are clearly overdispersed, but as we note in the text, the integration over possible values of the Poisson mean seems to be adequate to account for the major effects of this overdispersion in our application. Any disadvantages of the Poisson are easily offset by advantages in fitting efficiency, robustness, and interpretability. Indeed, we observe comparable performance between ACE and methods that use more sophisticated variance estimation methods across a large panel of cell lines (Figure S7). We now provide further explanation for these design decisions in the Discussion sectio*n.

3.	The authors use gamma parameters to connect the latent variables to observed counts. The gamma parameters are quite important, and the authors do not estimate them through MLE, but rather through a pre-processing step. The pre-processing estimations are reasonable; however, without any evidence/analysis, the paper claims this estimation is adequate compared to treating them as variables in MLE. Supportive evidence and/or analysis should be provided to justify the use of pre-processing estimates.

*We borrowed this heuristic estimator from the ‘normalization constants’ or ‘size factors’ used in DESeq2 (Love et al., 2014, Equation 1), which have proven to be adequate in this widely used tool. In initial experiments, we did not find the ACE to be very sensitive to the estimation method for these parameters, and we believe that the empirical performance of the program (Figures 3, 4, S5, S6, S7, S8, and S9) indicates that the heuristic method is sufficient for our purposes. However, we have edited the language in this section to be clearer about our rationale for the heuristic method, and to explain that we cannot guarantee that this method will perform as well as ML estimation in all settings.*

4.	The differential analysis is based on a likelihood ratio test of (Test times Control)/(combined Test and Control). This is a reasonable and adequate statistical test. It would be of interest to the readers to see this generalized to more than two group comparisons, e.g. borrowing ideas from intersection-union test (Casella, G. & Berger, R. L. Statistical Inference, 2002). This might be helpful for characterization of tumor types with high degree of heterogeneity in mutation profiles or other genomic features.

*We appreciate this suggestion. We believe a full derivation and implementation of multi-way testing is unfortunately beyond the scope of the current manuscript, but it would indeed be an interesting avenue to explore in future work. We have added brief mention of this point in the Discussion.*

5.	The comparison of this new method using the simulated data shows that JACKS, which utilizes a variational Bayes framework, outperforms the proposed ACE in the detection of essential genes. The differential assessment shows ACE outperforms existing methods and I consider this as a novel space as proposed by Hutton et al. However, there are two major concerns. First, there are other competitive algorithms such CRISPhieRmix and CERES that are not included in the Figs. 2 and 3. Hence, such additional methodologies should be included in the comparative evaluation. Second, while I appreciate the simulation results, method comparison should also cover real datasets such as Achilles (i.e. Broad DepMap) or Sanger CRISPR-Cas9 dependency datasets. To build controls, one can treat genes that are non-expressed across the majority of CCLE cell lines as negative controls, and known/published essential genes found from Achilles or DRIVE RNAi datasets as positive controls. To show the robustness of differential analysis, one could further categorize the positive controls into strong, modest, or weak groups according to the distribution of gene essentiality (for instance CERES scores) across all cell lines.

*As recommended, we performed a new benchmarking test on 42 liver and lung samples from the Broad DepMap data set (Figure S6-S8), this time including CRISPhieRmix and CERES along with the other methods. Genes were selected in three equal groups based on CCLE expression profiles in these samples, either globally expressed, unexpressed, or expressed relatively more highly in one tissue (differential gene set expression profile shown in Figure S6). ACE performs roughly as well as the best methods (Figures S8, AUC: CRISPhieRmix = 0.709, AFC = 0.705, ACE=0.701, JACKS=0.688, BAGEL=0.687, CERES=0.528), though most methods perform only modestly well on this benchmark, likely due to the disparity between gene expression levels and gene essentiality as evaluated by a knockout phenotype in our chosen gene set. The main outlier in this analysis is CERES, which shows considerably poorer performance than the other methods. This may reflect the strength of the hyperparameter λ, which was chosen to optimize CERES performance on uniformly essential and nonessential genes across all DepMap samples, and in this setting may be dampening weaker sample-specific signals of essentiality.*

*We also performed an additional analysis in Figure 4E and S9, ranking only MDM2 (a negative regulator of wildtype TP53) and CDKN1A (a positive regulator of wildtype TP53) against our positive and negative controls. ACE demonstrated the most reliable classification of both of these gold-standard genes, particularly with small numbers of samples per panel.*

*Finally, we added CRISPhieRmix to our comparison of methods based on simulated data (Figures 2 and 3), and found that ACE generally performed at least as well as this method, except for one case where CRISPhieRmix offered the best performance of all methods (Fig 3C with ɸ = 0.2). Because CERES heavily leverages copy-number information not included in our simulations, we were not able to include it in these comparisons.*

6.	In "Identification of Essential Genes" subsection, the authors employ pan-essential and non-expressed genes as positive and negative controls. These genes should be essential or non-essential regardless of cell line profiles. Hence, the ACE should not be limited to only NSCLC cell lines, rather expanding to all Achilles cell lines to evaluate ACE performance across a wider range of samples with more variability in essentiality levels. Moreover, there is no need to limit the analysis to only positive and negative control genes whereas the other genes can serve as another category (i.e. cell-line/subtype specific dependencies) to evaluate performance. In this category, the essentiality level should be lower than positive controls and potentially higher than negative controls.

*Following the reviewer’s suggestion, we now show the results of a benchmarking test based on a variety of lung- and liver-originating cell lines (Supplementary Figures S6-S8), as noted above. ACE performs competitively with the top methods compared, though the advantages of ACE are more clear-cut with a small number of samples on verified differentially essential genes, as we highlight in Figure 4E and S9.*

7.	In "Test for Non-Oncogene Addictions in TP53 Mutants" subsection (Fig. 5d), If all Achilles cell lines are used, a simple correlation between the dependency of TP53 and every other gene shows MDM2 is among top 5 well-correlated genes. Thus, there needs to be a method comparison to evaluate the sensitivity of differential genes with respect to the number of samples. This could elucidate in what cases ACE is strongly outperforms heuristic approaches.

*On further review, we found Figure 5D to be poorly designed and ineffective at illustrating our intended points. Therefore, we have removed this figure, and instead summarized our evidence for ACE’s sensitivity for differentially essential genes in Figures 4E and the associated supplemental figures. As noted, Figures S6-S8 show ACE’s relative performance for classifying differentially expressed genes as differentially essential across a large panel of lung and liver samples. As suggested by the reviewer, Figure 4E compares the performance of ACE and several other methods in identifying both canonical tumor suppressors and essential genes as a function of sample size.*

8.	The paper proposes the mRNA methyltransferases as new candidates based on a limited dataset, and it makes sense, and would seem easy to confirm/validate this finding in separate datasets. For instance, the Sanger data and/or another tumor type in Achilles datasets would be independent datasets that should clearly reproduce the results.

*Following the reviewer’s suggestion, we analyzed the essentiality of the mRNA methyltranferases in liver cell lines screened by Project Achilles, and in screens of NSCLC-derived cell lines from the Sanger’s contribution to the DepMap Consortium, Project Score, which uses a different CRISPR library. We found strong support for differential essentiality of these methyltransferases in NSCLC-derived cell lines, but the differential-essentiality feature did not appear to be present in liver-derived cell lines. This may be due to the over-representation of mutations in the Wnt pathway, specifically gain-of-function mutations in CTNNB1, dominating the essentiality signatures in wildtype TP53 samples in hepatocellular carcinoma (Ally et al. 2017). We now present this negative result in Figure S11, illustrating the relative change in effect sizes between TP53 wildtype and mutant sample panels in lung and liver, and address these findings in our revised Discussion.*

Minor Comments:

1. The paper uses MLE with a hierarchical model, however, the word "Bayesian" are often used, e.g. "hierarchical Bayesian model" throughout the paper. There is no Posterior estimation involved, and thus "Bayesian" should not be used.

*We thank the reviewer for this comment; while this statistical framework lends itself to a Bayesian extension, the actual software uses MLE for computational efficiency. We have therefore avoided use of the term “Bayesian” in our revision.*

2.	In "Benchmarking of Differential Essentiality Predictions" subsection, why not using limma on the gene-level values (i.e. w) inferred by JACKS to obtain differential genes? I am not sure the ratio of p-values for the test and control is an appropriate statistical test.

*The reviewer’s suggestion is a good one in principle, but we faced a challenge in using the reported ‘w’ values. Unlike CERES, which explicitly normalizes logFC data within each cell-line replicate (Meyers et al. 2017), JACKS reports a gene-knockout effect that is both gene- and condition/sample-dependent, but does not normalize ‘w’ within samples. As a result, direct comparison of ‘w’ across samples is problematic. However, the p-values reported by JACKS are more directly comparable because they reflect sample-dependent null distributions. We chose to use p-values rather than ‘w’ to leverage this normalization, and use the ratio of p-values when there is only one sample type per test/control panel, as in our simulations, and a Student’s t-test of the computed JACKS p-values when there are multiple sample types per panel. We have clarified these points in the Methods section.*

3.	Fig. 5d is confusing. I assume the asterisk next to MDM2 rank value shows the ranking when the test is wildtype TP53 while for the other rows the test is delta TP53? This needs a better representation since reader might think MDM2 is dependent in delta TP53. Also, it is worth showing where the CDKN1A's rank stands in wildtype TP53.

*As noted above, we have removed Figure 5D, and now use Figures 4E and S7-S9 to contrast ACE with CERES, and use Figure 5C to summarize the differential essentiality of the RNA methyltransferases in Project Score. We have also included CDKN1A in Figures 4E and S9.*

4.	The explanation of empiriCRISPR in Method section, the package to generate simulation data, is not clear.

*We have considerably expanded our discussion of empiriCRISPR in the Methods section, with the goal of making it clearer to the reader.*

5.	I could not find the ACE/empiriCRISPR packages. The package should be available, along with a manual/vignette.

*We apologize for the delay in the release of the software. We have now ensured that all of the software is publicly available, with clear and complete documentation.*

Reviewer #2

Major Comments:

1.	What are the applications or situations where the ACE framework would be most beneficial, above and beyond existing approaches? Would it for instance, be particularly useful for modelling low quality data where the fitness effect might be relatively modest, or sample variation is high? Would it be particularly useful for integrating data generated from different screens using different methodologies by accounting for additional experimental factors? Practical examples using 'real' data should be provided.

*We do indeed observe strong relative performance of ACE with very low numbers of samples, as illustrated by the substantial improvement in ranking tumor suppressor gene CDK1NA as well as by our results on simulated data (Figure 3). We have included additional Figures 4E and S9 showing ACE’s identification of two gold-standard differentially essential genes with increasing numbers of samples in publicly available DepMap data. In addition, we have included an analysis of differential essentiality classification using a larger set of differentially expressed genes as our validation population (Figures S6-S8). While genes identified using expression signatures have a much noisier signature of differential essentiality in CRISPR screens, reflected in weakened performance across all methods, ACE performs among the top methods examined. We also agree that our probabilistic framework is particularly well suited for integrating data across experiments, or for pooling data across classes of genes. Following the reviewer’s suggestion, we now highlight these advantages in the Discussion.*

2.	Copy number associated bias in gene fitness effects is a major confounder for Cas9 screens. Several methods have been developed to correct this effect (PMID: 29083409 and PMID: 30103702). A failure of ACE to systematically account for this effect is concerning and a limitation, and there is clearly an effect in their data (Figure S8a). Lung cancer is primarily driven my SNPs. Have the authors evaluated ACE in tissues types where copy number alterations are much more prevalent, such as breast or ovarian cancer cell lines, and much more likely to be have a pronounced confounding effect on the results?

*We appreciate the concern about biases associated with copy number, but several observations suggest that such biases are unlikely to be a major problem with ACE. While sample-specific scores find 10-40% of significantly differential genes are highly amplified without correcting for copy number (Meyers et al. 2017, Figure 5), we do not see a statistically significant correlation between gene copy number per panel and ACE’s measure of differential essentiality in the analyses presented in this paper, as illustrated in Figure S8b. Moreover, the expected effect of copy number variation on absolute essentiality scores, shown in Figure S8a, falls within the expected error demonstrated in our simulation study (Figure 2, ±0.1). However, we acknowledge that applications of ACE to small numbers of samples may amplify sample-specific effectors of gene essentiality, such as copy number. In the case of low sample numbers, we recommend that users include gene copy number among their guide-efficiency covariates (see Discussion).*

3.	Why have the authors excluded the number of base mismatches as a feature when determining sgRNA efficiency (and only included GC content)? This would seem important, especially when the mismatch is proximal to the PAM sequence.

*We are aware that there has been substantial recent progress on the problem of predicting sgRNA efficiency from a variety of genomic features, including the number of base mismatches. For simplicity, however, we chose to focus in our initial implementation on G+C content alone, which has consistently been shown to be one of the strongest features for predicting on-target guide efficiency (e.g., Doench et al. 2014, Doench et al. 2016, Gisler et al. 2019). We felt it was beyond the scope of this work to carry out a thorough evaluation of methods for sgRNA efficiency prediction in the context of ACE. Nevertheless, we have endeavored to make ACE as flexible as possible for leveraging more sophisticated efficiency prediction methods in future applications. One possible path forward, for instance, would be simply to substitute the efficiency score generated by the MIT’s CRISPOR tool in place of G+C content in ACE’s current model.*

4.	Related to Figure 4. How does the performance of ACE compare to JACKS or BAGEL for the identification of essential genes when using the DepMap data? Also, for Figure 4a, can the authors comment on why so many genes appear to be classified as having decreased estimated essentiality?

*Following the suggestion by this reviewer and others, we now compare several methods, including JACKS and BAGEL, on DepMap data in Figures 4E, S7, S8, and S9. As noted above, several methods perform similarly in Supplementary Figures S7 and S8, but the classification of differential essentiality is constrained by the quality of our set of ‘differentially essential’ genes. As shown in Figure 4E, ACE performs as well or better than JACKS or BAGEL on MDM2 and CDKN1A, especially at small numbers of samples. These particular genes were chosen for their properties as canonical examples of TP53-dependent differential essentiality, negative and positive regulators respectively, and further recommended by Reviewer 1.*

*In Figure 4A, the points corresponding to negative essentiality estimates highlighted by the reviewer are visually pronounced, owing to the structure of the plot, but the vast majority are not significantly different from zero, as indicated by their appearance below the horizontal dashed line in that figure. With real data, the essentiality statistic often takes a negative value, owing to random fluctuations in cell growth, relative sgRNA abundance, and sequencing read counts, but these negative values are generally not significant. The genes with significant negative essentiality, such as POU1F1 on the far left, have been mis-classified as negative controls due to low expression levels, but may actually have growth suppressing activity (Scully et al. 2000). A note has been added to the manuscript for clarity.*

5.	The authors demonstrate that ACE can detect differential essentiality in test and control samples , using TP53 and KRAS in NCSLC as examples. The number of lung cell lines screened is large (n = 92) and these mutations are very prevalent in this tissue. How does ACE perform when working with smaller sample sizes which are typically the case for other tissue lineages and mutational events?

*As noted, we have now added Figure 4E to illustrate ACE’s sensitivity for differential essentiality as a function of the number of samples. We find that ACE has particularly good power in comparison to other methods with small numbers of samples. Note also that ACE easily detected the differential essentiality of our candidate RNA methyltransferases in a much smaller sample panel comparison using Project Score data (Figure 5C).*

6.	The authors argue the identification of RNA methyltransferases as a differential essentiality in TP53 mutant cells demonstrates the advantages of the ACE framework. While this observation is interesting, the finding is not validated with further replication studies to confirm the differential dependency, and no mechanistic insight is provided. Further, it is unclear why this difference is observed, except that they are using a different analytical framework. Thus, while the observation is interesting, and there are circumstantial evidence supporting the observation, it does not provide evidence that one approach is superior or more informative than the other without further insight.

*We thank the reviewer for suggesting further replication studies, and have further provided evidence of ACE’s relative performance on known differentially essential genes in Figure 4E. It is difficult to compare method performance outside of these strongly differential examples in real data, as illustrated in Figures S6-8, due to the challenge of identifying differentially essential genes from non-CRISPR methods, and thus must rely upon our simulation study in Figure 3 for quantitative assessment of differential essentiality identification. We have further supported our identification of the TP53-dependent essentiality of the RNA methyltransferases with new results in the Project Score CRISPR screens in Figure 5C. As reviewers #1 and #3 have pointed out, further biological validation is out-of-scope for our current Software paper. Further experiments are certainly required to conclusively demonstrate this phenotype, and we have added to our discussion to emphasize this point.*

7. Large studies reporting CRISPR screens in cell line panels from the Sabatini lab (28162770) and Sanger Institute (PMID: 30971826) should be cited in the introduction.

*We thank the reviewer for bringing these references to our attention, and we now cite them in the manuscript. We had briefly mentioned the Sabatini lab screenings as an example of screens with both initial and depleted sequencing available (source 15 in our previous submission), but we now highlight this reference more clearly. A citation of the Sanger Institute’s contribution to the DepMap Consortium, which provides the data in our new Figure 5C, was previously omitted in error.*

Reviewer #3

Major Comments:

1.	As biological validation is out-of-scope of this computational/methodological study, the authors should strive to further substantiate their findings by additional analyses. The analysis presented in the manuscript was carried out on lung adenocarcinoma cell lines. (a) Does MDM2 turn essential only in p53-wt background in cell lines of other cancer types? (b) Do METTL14/16/3 show enhanced essentiality in p53-Mut background in cell lines of other cancer types? The indication of these METTLs as candidates for non-oncogene addiction in p53-Mutant cancer is very interesting. However, the support given for this observation is not strong (their expression levels (Fig 5c and FigS7)). Finding this effect in additional cancer types can significantly strengthen this result.

*Following this suggestion and similar ones from the other reviewers, we have examined both liver samples in the Project Achilles’ database, chosen for its relatively abundant and homogenous samples, and CRISPR screens in lung-derived cell lines generated by the Sanger Institute for Project Score. While METTL3/14/16 showed no differential essentiality in liver samples, we observed a very strong signature of differential essentiality in the lung-derived cell lines, despite the use of a different CRISPR library and a relatively small sample size. We thank the reviewer for suggesting this analysis, as it suggests this differential essentiality is lung-specific. As we now mention in the Discussion, additional experiments will ultimately be required to validate this finding.*

2.	The usefulness of ACE package is largely dependent on the availability of a detailed user-friendly manual. I couldn't find ACE (nor empiriCRISPR) at the given link.

*We apologize for the delay in the release of the software. We have now updated our repositories and documentation, and made them publicly available. We have also archived the software version at time of submission using Zenodo, as requested by the editor.*

3.	Fig. 4a. The scatter of the negative controls shows a branch to the right-top above significance threshold. Are these genes characterized by any specific biological annotation? Are they in fact true positives?

*We found these negative control outliers to have a mix of biological function. Of the top 5 (DUX4, GOLGA6L1, SPDYE3, PPIAL4E, SPDYE16), DUX4 is located within a repeat array but is nevertheless essential as a transcriptional regulator, the SPDYE genes are necessary for completion of the cell cycle, GOLGA6L1 has limited documentation, and PPIAL4E is detected in Northern blot analysis in most tissue. Overall, we find it most likely that these genes are misclassified as ‘negative controls’ by the gene expression data we used. We now mention this point in the text.*

4.	Fig S8. The title argues that ACE essentiality estimates avoid copy number bias. However panel (a) shows a significant overall correlation between copy number and ACE essentiality.

*While the slope of the line in what is now Figure S9B appears positive, it is not significantly different from zero. Thus, we do not find a statistically significant correlation between copy number and ACE’s test statistic for differential essentiality, which was used to nominate our candidate methyltransferases. We have altered the figure and legend to emphasize the absence of statistical significance. However, we acknowledge copy number could more strongly influence results for panels with few sample types; we have expanded our discussion to address these cases, for which we recommend supplying target gene copy number as a guide efficiency feature.*

**Second round of review**

**Reviewer 1**

The authors adequately addressed comments regarding methodology and computation. The addition of new methods for benchmarking and new updates to the text are reasonable. However, there remain concerns regarding the analytics over real data. First, the authors use liver and lung samples from the Broad DepMap datasets for differential essentiality benchmarking (Supplementary Figures S7 and S8) where the majority of methods, including ACE, perform the same. I am not clear as to why limiting the analysis to only lung vs liver, where more cancer type pairs can be included to expand the analysis and to make a broader conclusion. Second in Figure 4E where lung data are utilized, the authors show the advantage of ACE specifically with the low number of samples. Nonetheless, this advantage is limited to just one case (CDKN1A). I do believe a more comprehensive analysis to show a substantial power in detecting differential genes in the context of real data is needed.

**Authors Response**

**Point-by-point responses to the reviewers’ comments:**

*We again thank the editors and reviewers for their thoughtful and constructive attention to our manuscript.*

*The reviewers were apparently generally satisfied with our initial round of revisions, but Reviewer #1 did have one final suggestion, which the editor asked us to address:*

Reviewer #1: The authors adequately addressed comments regarding methodology and computation. The addition of new - methods for benchmarking and new updates to the text are reasonable. However, there remain concerns regarding the analytics over real data. First, the authors use liver and lung samples from the Broad DepMap datasets for differential essentiality benchmarking (Supplementary Figures S7 and S8) where the majority of methods, including ACE, perform the same. I am not clear as to why limiting the analysis to only lung vs liver, where more cancer type pairs can be included to expand the analysis and to make a broader conclusion. Second in Figure 4E where lung data are utilized, the authors show the advantage of ACE specifically with the low number of samples. Nonetheless, this advantage is limited to just one case (CDKN1A). I do believe a more comprehensive analysis to show a substantial power in detecting differential genes in the context of real data is needed.”

*In response to Reviewer #1’s comments, we have greatly expanded the real data analysis shown in Figure 4 and Supplementary Figures S7 and S8 to analyze the essentiality of 19 known differentially essential genes across 14 genetic backgrounds in 5 tissue types (ovary, blood, colorectal, skin, and lung). We have included 3 randomly selected replicates of cell lines in each tissue category for each class of genetic background to limit our test performance in the low-sample regime. For comparison, we have also summarized the performance of several other methods across these same gene and tissue combinations in Figure 4F, and provided detailed ACE results in Figure S10. We found that ACE consistently ranked these known differentially essential genes above uniform controls at a higher rate than other methods, even in the low-data regime. We believe these additional analyses clearly demonstrate the advantages of ACE when working with real data.*

**Third round of review**

**Reviewer 1**

The authors adequately addressed my comments. The new additions demonstrate the advantages of ACE over existing methods.
